# Converting non-neutralizing SARS-CoV-2 antibodies into broad-spectrum inhibitors

**DOI:** 10.1038/s41589-022-01140-1

**Published:** 2022-09-08

**Authors:** Payton A.-B. Weidenbacher, Eric Waltari, Izumi de los Rios Kobara, Benjamin N. Bell, Mary Kate Morris, Ya-Chen Cheng, Carl Hanson, John E. Pak, Peter S. Kim

**Affiliations:** 1 Sarafan ChEM-H, Stanford University, Stanford, CA USA; 2 Department of Chemistry, Stanford University, Stanford, CA USA; 3 Chan Zuckerberg Biohub, San Francisco, CA USA; 4 Stanford Immunology Program, Stanford University School of Medicine, Stanford, CA USA; 5 Department of Molecular and Cellular Physiology, Stanford University School of Medicine, Stanford, CA USA; 6 California Department of Public Health, Richmond, CA USA; 7 Department of Biochemistry, School of Medicine, Stanford University, Stanford, CA USA

**Keywords:** Proteins, Infectious diseases, Infectious diseases

## Abstract

Omicron and its subvariants have rendered most authorized monoclonal antibody-based treatments for severe acute respiratory syndrome coronavirus 2 (SARS-CoV-2) ineffective, highlighting the need for biologics capable of overcoming SARS-CoV-2 evolution. These mostly ineffective antibodies target variable epitopes. Here we describe broad-spectrum SARS-CoV-2 inhibitors developed by tethering the SARS-CoV-2 receptor, angiotensin-converting enzyme 2 (ACE2), to known non-neutralizing antibodies that target highly conserved epitopes in the viral spike protein. These inhibitors, called receptor-blocking conserved non-neutralizing antibodies (ReconnAbs), potently neutralize all SARS-CoV-2 variants of concern (VOCs), including Omicron. Neutralization potency is lost when the linker joining the binding and inhibitory ReconnAb components is severed. In addition, a bi-functional ReconnAb, made by linking ACE2 to a bi-specific antibody targeting two non-overlapping conserved epitopes, defined here, shows sub-nanomolar neutralizing activity against all VOCs, including Omicron and BA.2. Given their conserved targets and modular nature, ReconnAbs have the potential to act as broad-spectrum therapeutics against SARS-CoV-2 and other emerging pandemic diseases.

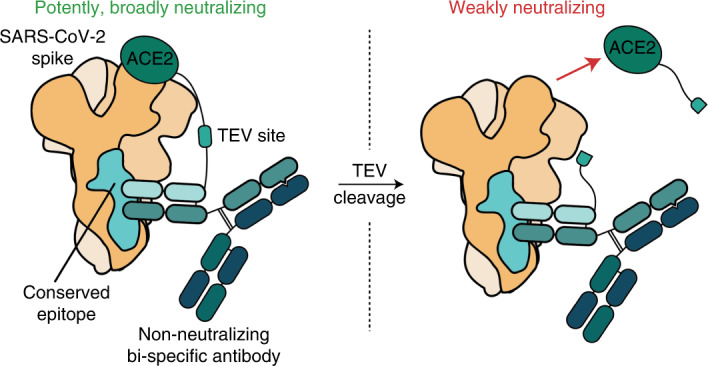

## Abstract (web summary)

SARS-CoV-2 spike-directed, non-neutralizing antibodies were converted into broad-spectrum inhibitors by conjugation to the SARS-CoV-2 receptor, ACE2, resulting in fusion proteins that target all SARS-CoV-2 variants of concern tested.

## Main

The emergence of the Omicron variant has rendered six of the seven^1,2^ clinically available monoclonal antibodies (mAbs) essentially ineffective against SARS-CoV-2; only sotrovimab retains robust neutralizing activity against Omicron^2,3^. These clinical mAbs all target the receptor binding-domain (RBD)^1^ of the spike protein and were selected for their neutralizing potency against Wuhan-Hu-1 SARS-CoV-2. The six mAbs besides sotrovimab target non-conserved (variable) regions of the RBD^4–7^ and prevent interaction with its receptor, ACE2 (refs. ^6,8,9^). Sotrovimab, a derivative of the mAb S309 (ref. ^10^), was initially isolated from a survivor of SARS-CoV-1, so its epitope in the RBD is more highly conserved^11^, although in vitro escape mutations have been identified^5^. Moreover, since this article has been in review, sotrovimab has lost significant activity against the recent BA.2 variant^12^. This has necessitated authorization of a new mAb, bebtelovimab, which is capable of neutralizing BA.2 (ref. ^13^).

**Fig. 1:**
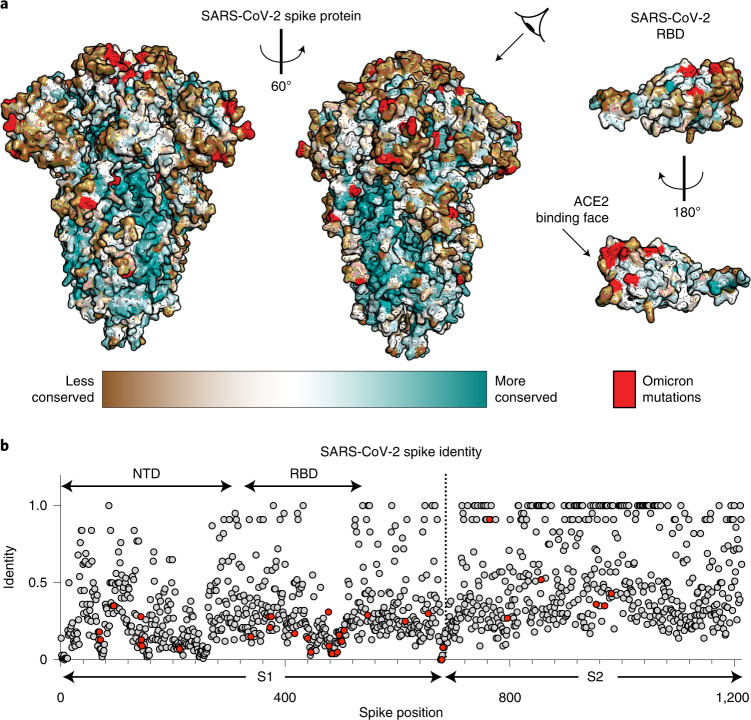
Conservation of the SARS-CoV-2 spike protein. **a**, Sequence conservation from 44 related spike proteins overlaid on the SARS-CoV-2 spike protein structure (left) and the SARS-CoV-2 RBD (right; residues 319–541) (PDB ID: 6VXX) identifies a highly conserved patch in S2. Color gradient is a step gradient of conservation containing nine total steps of sequence conservation identified from the ConSurf database; gradient is shown on the bottom. **b**, Sequence identity for all residues in the SARS-CoV-2 spike protein compared to a set of 44 related coronavirus spike proteins shows higher conservation in the S2 relative to the S1. A value of 1.0 means perfect identity across all compared coronavirus proteins. RBD and NTD domains of SARS-CoV-2 spike are labeled on the top; S1 and S2 domains are labeled on the bottom.

The spike protein is large (~450 kDa as a trimer) and contains extensive regions that are extremely highly conserved (Fig. 1a). Some residues on the spike that are distant from the RBD have near-perfect sequence identity within related coronaviruses (Fig. 1b). Presumably, these regions are highly conserved because they are required for viral activity (for example, membrane fusion)^14^. Although the spike protein of Omicron has a much larger mutational profile than that of previous VOCs^15^—with 36 total mutations, 15 being in the RBD^2,16^—the highly conserved epitopes remain largely unaltered (Fig. 1)^2,16^.

In other viral spike proteins—for instance, influenza hemagglutinin^17–19^—highly conserved epitopes outside of the receptor-binding region are targets of potent broadly neutralizing antibodies (bnAbs). However, despite the heightened interest sparked by the global pandemic, the search for bnAbs against betacoronaviruses has been largely disappointing. Although one conserved helical epitope at the base of the spike protein has been shown to elicit rare mAbs with relatively broad neutralizing activity, their potency is often weaker than RBD-directed neutralizing Abs^20–22^. Furthermore, neutralizing N-terminal domain (NTD)^23^ antibodies have been identified, but their epitopes are not highly conserved.

Indeed, available evidence suggests that conserved regions outside the RBD generally elicit non-neutralizing mAbs^21,24–29^. We hypothesized that we could generate potent, broad-spectrum inhibitors by modifying existing non-neutralizing antibodies, which target highly conserved epitopes on the spike protein, to also contain a receptor-blocking component. Due to the conservation of their epitopes, such inhibitors would potentially be broadly neutralizing.

Here we introduce ReconnAbs (pronounced ‘recon-abs’), a novel class of therapeutic proteins in which non-neutralizing antibodies that target highly conserved, non-RBD epitopes are tethered to the ACE2 receptor, which otherwise has low intrinsic affinity and neutralizing potency. The cross-reactive, non-neutralizing antibodies were identified in a two-step process. First, we analyzed the phylogenetic trees of a collection of SARS-CoV-2 antibodies and eliminated those that are likely to bind the RBD. Then, similarly to the development of sotrovimab^11^, we determined which of these non-RBD antibodies bound to the SARS-CoV-1 spike protein. We predict that ReconnAbs will have increased potency due to the increase in effective concentration of each component^30^, as has been shown previously for bi-specific antibody fusions^31^ and antibody–ACE2 fusions^32^. More importantly, ReconnAbs are predicted to have increased broad-spectrum activity by targeting highly conserved, non-RBD epitopes on spike. We demonstrate that ReconnAbs show neutralizing activity against all SARS-CoV-2 VOCs tested, including Omicron and BA.2. Furthermore, a bi-specific ReconnAb containing two non-neutralizing antibodies with non-overlapping epitopes fused to ACE2 confers sub-nanomolar neutralization against all VOCs tested. Our findings reveal the benefit of repurposing highly cross-reactive, non-neutralizing antibodies to create a new class of broad-spectrum anti-viral agents.

## Results

### Cross-reactive antibody identification

To first profile the landscape of non-neutralizing antibodies, we produced a library of SARS-CoV-2 spike-binding antibodies not directed against the RBD. To ensure library diversity, we first curated the publicly available SARS-CoV-2 antibody repository Coronavirus Antibody Database (CoV-AbDab)^33^ for sequences specifically from Coronavirus Disease 2019 (COVID-19) convalescent donors that bound to the spike protein outside of the RBD. From this limited set of 696 antibody sequences, we generated phylogenetic trees for both the antibody heavy chain (HC) and light chain (LC). We constructed phylogenies using amino acid sequences of full-length V-genes and the CDR3 region. We also included one allele of each germline V-gene into the phylogenies to provide context for germline diversity of the antibody dataset.

**Fig. 2:**
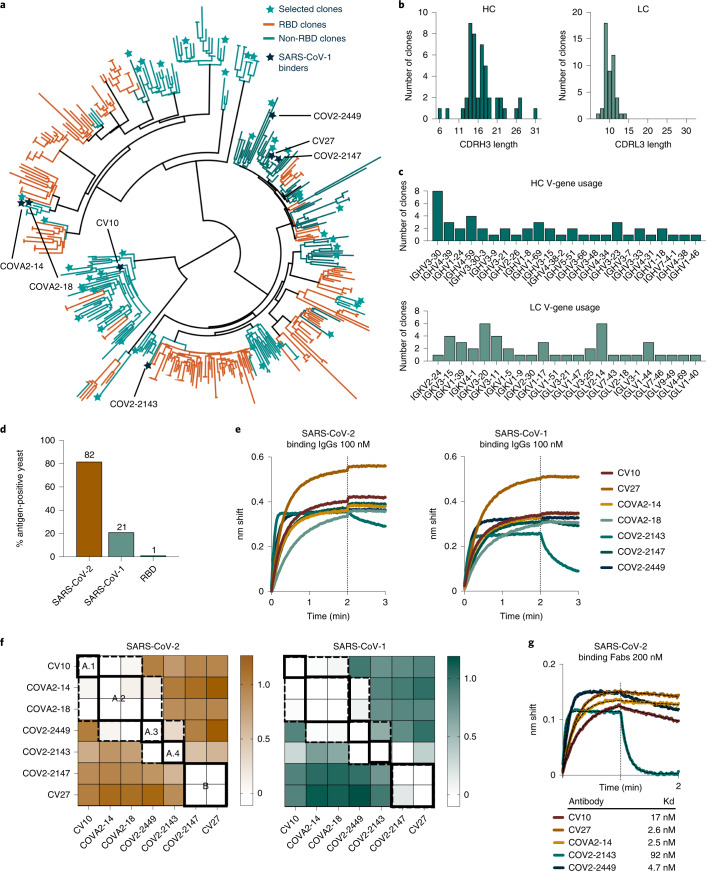
Non-RBD antibodies, selected to prioritize diversity, were used to identify non-RBD SARS-CoV-2 antibodies that bind SARS-CoV-1, a surrogate for epitope conservation. **a**, A phylogenetic tree of 422 HC sequences, from our curated library of 696 anti-SARS-COV-2 spike antibodies, generated using Geneious Prime. Germline alleles are not shown. Forty-eight selected clones are shown as stars. Histograms of the HC and LC (**b**) CDR3 lengths and (**c**) V-gene usage from the 48 selected non-RBD clones indicated in **a**. **d**, A binding profile of the scFv-yeast library produced from the sequences identified in **a** and Extended Data Fig. 1. **e**, BLI binding of identified cross-reactive clones expressed as IgGs at 100 nM to SARS-CoV-2 spike (left) or SARS-CoV-1 spike (right). **f**, BLI competition binding assay of the seven cross-reactive antibodies binding to SARS-CoV-2 (left) and SARS-CoV-1 (right). White indicates no binding of the tested antibody, indicating that the antibodies compete for binding. Antibodies that compete are surrounded by dotted lines; unique competition groups are surrounded by solid lines. The five unique competition groups are labeled on the SARS-CoV-2 binding competition map. Sites A.1–A.4 are indicated as an overlapping supersite. Loading antibodies are indicated in columns, and competing antibodies are indicated in rows. **g**, Binding of antibody Fab fragments at 200 nM against SARS-CoV-2 spike. Hashed lines show K_D_ fit determined using Prism.

**Extended Data Fig. 1:**
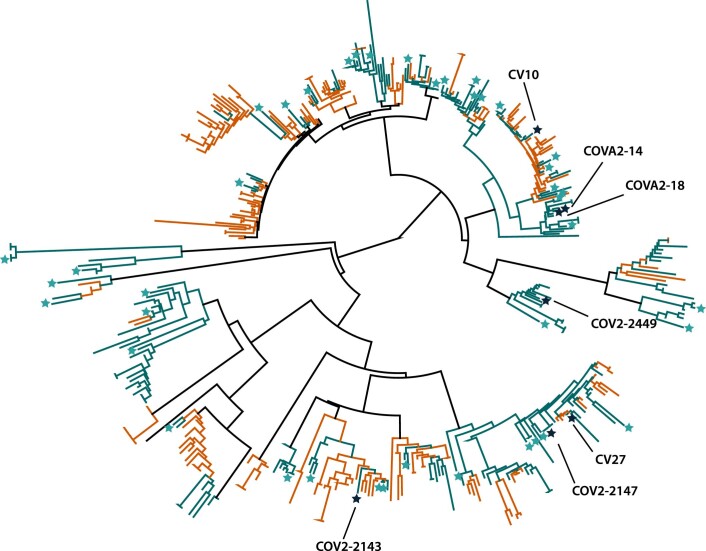
The non-RBD library was selected to prioritize diversity. A phylogenetic tree generated using Geneious Prime of 436 light chain sequences from a curated library of 696 anti-SARS-COV-2 spike antibody sequences. Labels same as Fig. 1. Germline alleles are not shown. Antibodies denoted with names were cross-reactive for SARS-CoV-1.

We compiled non-RBD-binding antibodies, focusing specifically on the clustering of the non-RBD-binding sequences within the HC phylogenetic tree (Fig. 2a). We identified distinct clusters on the HC and LC phylogenetic trees and chose 48 diverse sequences spread throughout the trees (Fig. 2a and Extended Data Fig. 1). These included at least one antibody sequence from all clusters containing four or more non-RBD-binding antibodies. The sequences we chose based on their HC sequences also showed diversity in the LC phylogenetic tree (Extended Data Fig. 1). These 48 clones display a range of CDRH3 and CDRL3 lengths (Fig. 2b) and use an array of V-genes in both the HC and LC (Fig. 2c), further confirming their diversity.

**Extended Data Fig. 2:**
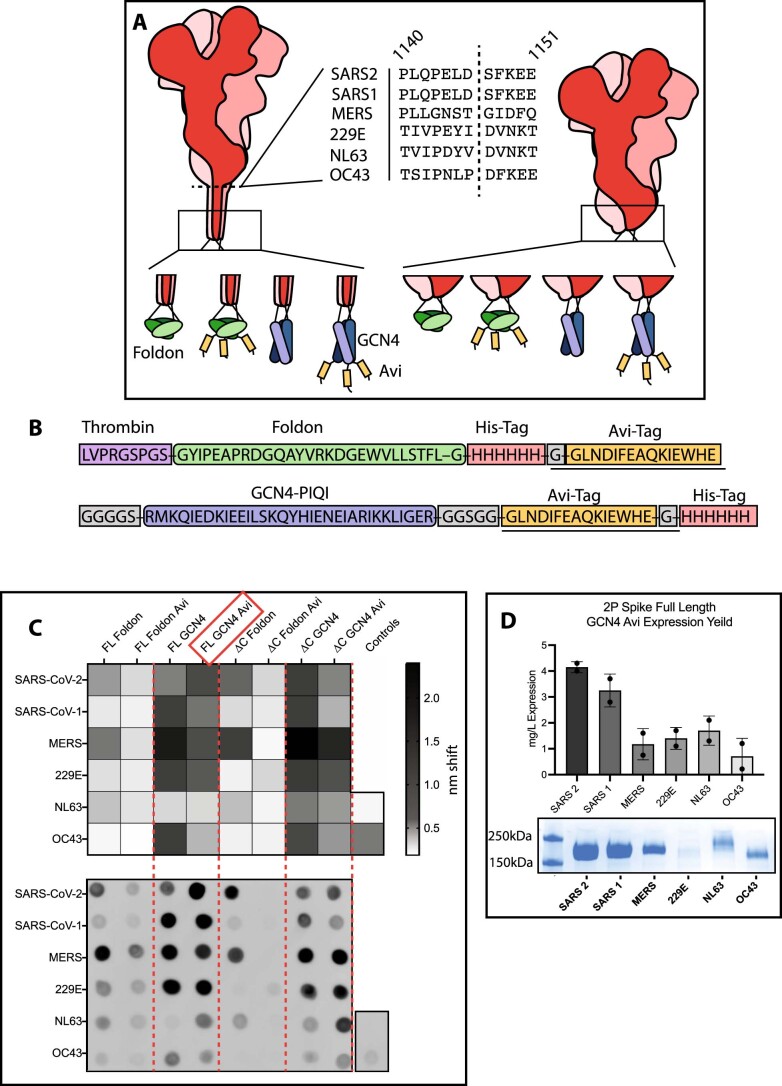
hCoV proteins expression is optimized with a GCN4 tag. (A) A schematic representation of constructs tested for expression. 6 hCoV spike proteins, either FL or truncated, tested with 4 different C-terminal trimerization domains and tags. ∆C truncations shown in the middle, SARS-CoV-2 spike numbering. (B) linkers tested. For those without Avi tags, the underlined portions were removed. (C) (top) BLI binding responses from isolated Expi supernatants binding to His 1 K octet tips. Higher response corresponds to higher protein expression. (bottom) An anti-his tag dot blot assay of the supernatants tested in the BLI binding. Dot blot shows good correspondence with the BLI binding. Influenza hemagglutinin used as a positive control, mock transfection as a negative control. (D) Yield determined for full length 2 P hCoV constructs containing the GCN4-Avi-His tag from a duplicate experiment of 100 mL transfections, error bars denote standard deviation.

To determine which of these 48 non-RBD-binding antibodies target highly conserved epitopes, we used binding to the SARS-CoV-1 spike as a surrogate for epitope conservation. We designed the 48 single-chain variable fragment (scFvs) constructs by fusing the antibody HC and LC variable regions to the yeast surface protein Aga2p to enable yeast surface display. To profile the scFv panel, we optimized production of biotinylated SARS-CoV-2 and other human coronavirus (hCoV) spike proteins (Extended Data Fig. 2a–d) and produced biotinylated versions of the SARS-CoV-2 and SARS-CoV-1 spike proteins. These were used to probe the yeast library by fluorescence flow cytometry (Fig. 2d). The complete 48-member library showed robust (82%; Fig. 2d) staining with the SARS-CoV-2 spike, consistent with the original antibody collection having been isolated from SARS-CoV-2 convalescent donors. The library had reduced (21%; Fig. 2d) staining with the SARS-CoV-1 spike. Consistent with the intention of the library, no clones bind to the RBD of SARS-CoV-2 (Fig. 2d).

**Extended Data Fig. 3:**
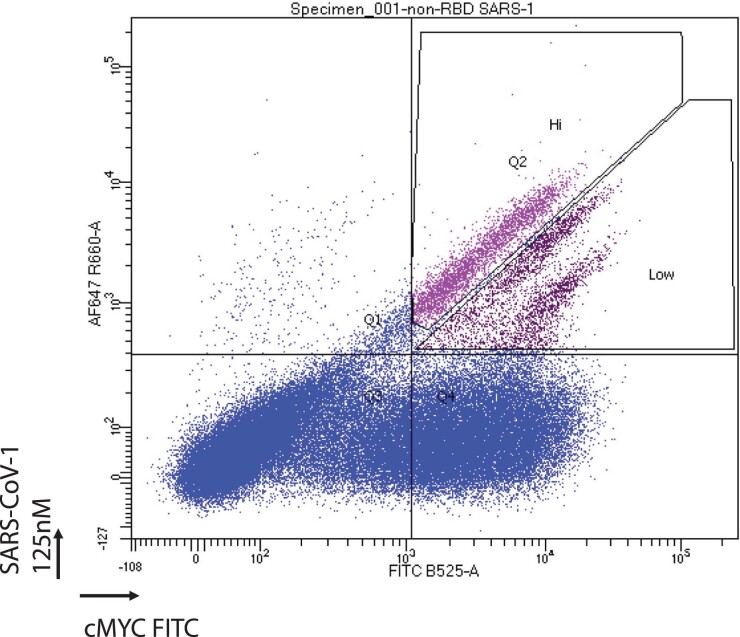
The non-RBD yeast library was sorted for SARS-CoV-1 binding antibodies. The gating scheme utilized to collect cross-reactive SARS-CoV-2 and SARS-CoV-1 binding yeast. Both the high and low gates were combined following the sort.

**Extended Data Fig. 4:**
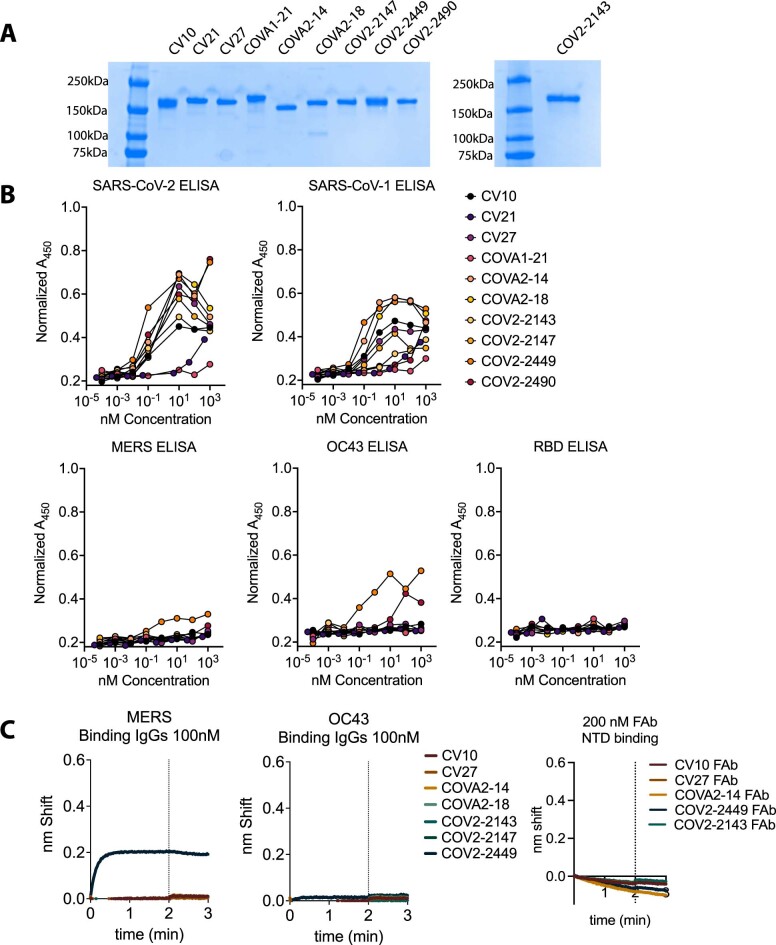
Antibodies isolated from yeast sorts are cross-reactive. (A) SDS-page analysis of of IgG proteins made from clones identified in the SARS-CoV-1 FACS sorts. MW ladders are in the left-most lanes of the two gels. (B) ELISA binding of IgG proteins made from scFv clones identified by FACS sorting for SARS-CoV-1 binding. Biotinylated hCoV antigens were plated and dilutions of IgGs were tested for binding. Normalized A_450_ calculated by adjusting for pathlength. Except for COV2-2490, CV21, COVA2-18 all IgGs bind to SARS-CoV-2 and SARS-CoV-1. (C) BLI binding for the 7 identified SARS-CoV-1 cross-reactive clones against MERS (left) or OC43 (middle) spike proteins. Only COV2-2449 shows any binding affinity for MERS or weakly to OC43 spike proteins. No Fabs tested bind to the SARS-CoV-2 NTD, consistent with its low sequence conservation between SARS-CoV-2 and SARS-CoV-1.

**Extended Data Fig. 5:**
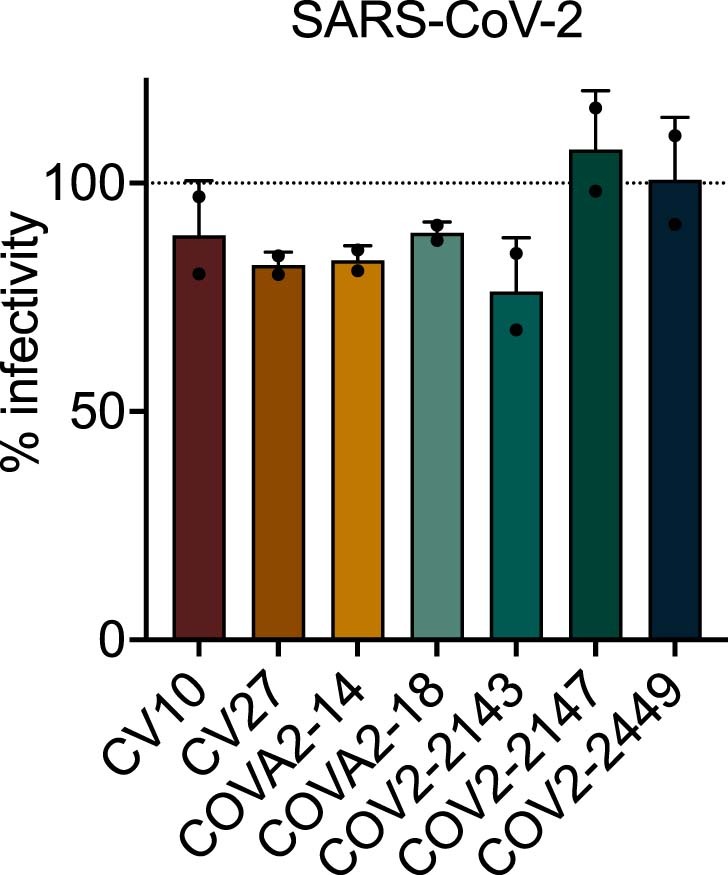
Antibodies isolated from yeast sorts are non-neutralizing. Single dilution (100 nM) neutralization against SARS-CoV-2 Wuhan-Hu-1 for the 7 identified cross-reactive antibodies. All antibodies show no neutralization at a high concentration of 100 nM. Error bars denote standard deviation.

Having identified 48 antibodies that bind outside the RBD, we next selected those that bind to highly conserved regions of the spike protein. To do this, we used fluorescence-activated cell sorting (FACS)^34^ with the SARS-CoV-1 spike protein as bait (Extended Data Fig. 3) and identified ten sequences. We confirmed by ELISA that the corresponding full-length IgG antibodies (Extended Data Fig. 4a) bind to both SARS-CoV-2 and SARS-CoV-1 spike proteins (Extended Data Fig. 4b). Of the ten, seven clones were strong SARS-CoV-1 binders, confirmed by biolayer interferometry (BLI) (Fig. 2e). One clone, COV2-2449, also binds MERS and OC43 spike proteins (Extended Data Fig. 4b,c). No clones bind to the NTD (Extended Data Fig. 4c). Consistent with previous reports^33,35–38^, these antibodies were non-neutralizing in our assay (Extended Data Fig. 5).

We used BLI to characterize the epitopes of these seven antibodies in a binding competition assay. We loaded each antibody onto either SARS-CoV-2 or SARS-CoV-1 spike proteins, and then we tested for subsequent binding of each of the other antibodies (Fig. 2f). The results suggest that there are five primary epitopes, of which four are in a partially overlapping supersite (Fig. 2f), likely corresponding to the extensive, continuous patch of highly conserved residues on the spike protein surface (Fig. 1a). Two sets of antibodies had indistinguishable epitopes: the pair^38^ of COVA2-14 and COVA2-18 and the pair of CV27 and COV2-2147. This result is consistent with the phylogeny, which shows the antibodies in the two pairs clustered very closely together (Fig. 2a and Extended Data Fig. 1). The identification of five unique epitopes from the seven selected antibodies highlights the diversity in the initial starting library.

### scFv-based ReconnAb development

**Fig. 3:**
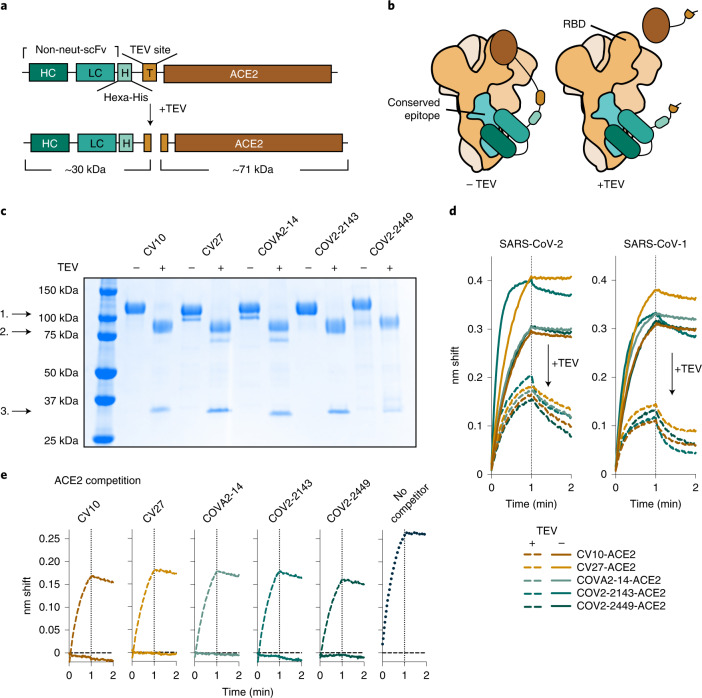
scFv-based ReconnAbs tether ACE2 to cross-reactive, non-neutralizing antibodies. **a**, A schematic of an scFv-based ReconnAb protein before and after TEV cleavage. Estimated molecular weights of cleavage products are shown beneath both. **b**, Schematic of ReconnAb activity and the dependence on the tether. The scFV binds to a conserved site, and then ACE2 interacts with the RBD. Upon TEV cleavage, the ACE2 has lower apparent affinity and does not bind the RBD (right). Conserved sites are shown as teal; the remainder of the spike monomers are shown as tints of brown and ACE2 as dark brown. **c**, SDS-PAGE demonstrates that ReconnAbs are readily cleaved by TEV. 1. Full-length ReconnAb; 2. ACE2; 3. scFv. **d**, BLI binding of uncleaved and TEV-cleaved ReconnAbs to either SARS-CoV-2 spike (left) or SARS-CoV-1 spike (right) shows a reduction in binding upon TEV cleavage. **e**, BLI binding of hFc-ACE2 to SARS-CoV-2 spike, which has been pre-associated with ReconnAbs either uncleaved (solid lines) or cleaved (hashed lines), shows that TEV-cleaved ReconnAbs do not compete with hFc-ACE2 binding. Binding of hFc-ACE2 without competitor is shown on the right (dotted line).

**Extended Data Fig. 6:**
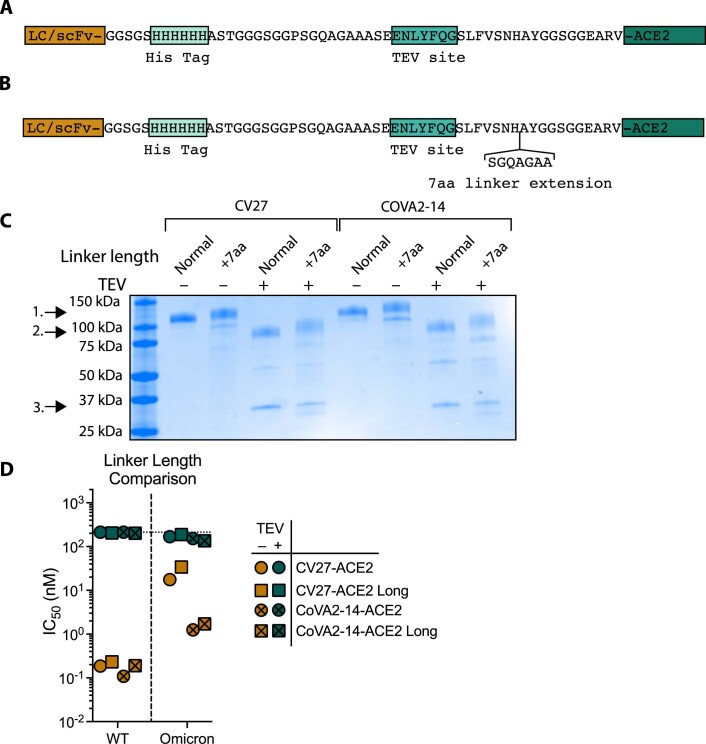
A longer linker length does not impact neutralization potency of ReconnAbs. (A) The linker used to tether ACE2 to either the C-terminus of the scFv or C-terminus of the COV2-2449 LC. The linker was designed to contain a Hexa-His tag for purification and a TEV site to facilitate proteolysis. (B) A longer linker length did not alter ReconnAb activity. One scFv (targeting either epitope A or B – as in Fig. 2f) was mutated to contain a linker that was 7aa longer than the previous study (B). An SDS-Page gel demonstrates that these proteins proteolyzed identically to those with the shorter linkers (C). The neutralization potency was not impacted by the increased linker length (D). Data shown is the average of 2 neutralization experiments.

We selected five antibodies, one from each described epitope (Fig. 2f,g), and converted these non-neutralizing, cross-reactive antibodies into ReconnAbs by fusion to the ACE2 ectodomain, as the receptor-blocking component of the ReconnAb design. We designed the linker to be long enough to allow for simultaneous binding of both ACE2 to the RBD and the scFv, regardless of epitope, to the spike S2 domain (Fig. 3a and Extended Data Fig. 6a). We joined the C-terminus of the scFv to the N-terminus of ACE2, because the N-terminal residue of the ACE2 ectodomain is adjacent to the SARS-CoV-2 RBD when bound. We also incorporated within the linker a hexa-histidine tag for purification and a TEV protease site to enable assessment of ReconnAb activity when its binding and inhibitory components are separated (Fig. 3a and Extended Data Fig. 6a). We anticipated that ReconnAbs would bind to both a highly conserved site on the spike protein and, simultaneously, to the RBD through the ACE2 domain (Fig. 3b). However, if cleaved at the TEV site, the intrinsically low-affinity ACE2 domain would not benefit from the affinity of the non-neutralizing antibody (Fig. 3b).

We expressed and purified the five ReconnAbs and used gel electrophoresis to confirm that TEV cleavage separated the ACE2 and scFv components (Fig. 3c). BLI experiments showed that TEV cleavage of the ReconnAb proteins reduced binding to both SARS-CoV-2 and SARS-CoV-1 spike proteins (Fig. 3d), consistent with the lower affinity of monomeric ACE2 (ref. ^39^). We then investigated the ability of the ReconnAbs to block ACE2 binding to the SARS-CoV-2 spike protein. ACE2 competition is often used as a surrogate for neutralization, as preventing ACE2 binding prevents the virus from interacting with target cells. Indeed, uncleaved ReconnAbs show substantial interference with binding of an Fc version of human ACE2 (hFc-ACE2), whereas TEV-cleaved ReconnAbs do not (Fig. 3e).

**Fig. 4:**
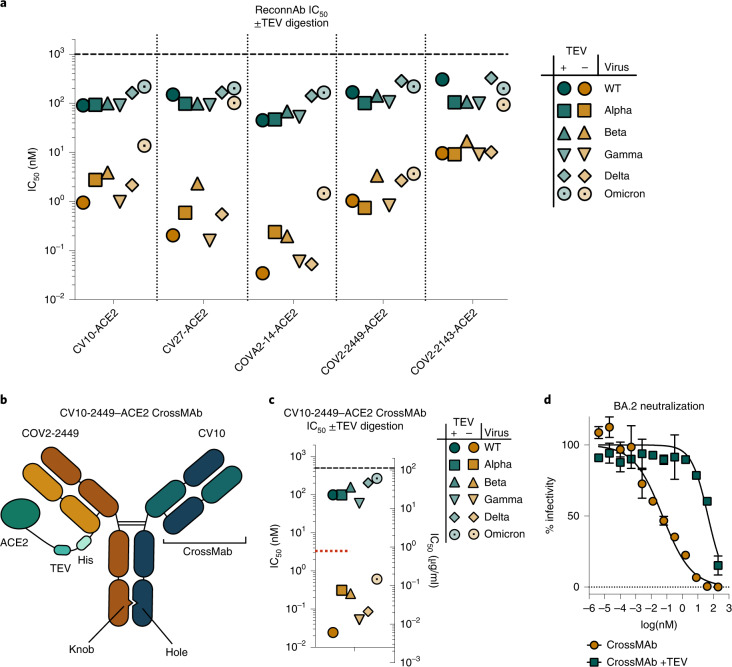
ReconnAbs show broad-spectrum neutralization of SARS-CoV-2 VOCs. **a**, Intact scFv-based ReconnAbs (orange) show potent neutralization of SARS-CoV-2 VOCs. Inhibition is markedly reduced upon TEV cleavage (teal). Pseudoviral IC_50_ for ReconnAbs against a range of SARS-CoV-2 VOCs with and without TEV cleavage. IC_50_ values shown are the average of two independent experiments. **b**–**d**, A bi-functional IgG ReconnAb shows potent neutralization of SARS-CoV-2 VOCs. **b**, A schematic representation of the CV10-2449–ACE2 CrossMAb indicates linkage of ACE2 and bi-functional Fab arms. **c**, Pseudoviral IC_50_ for CV10-2449–ACE2 CrossMAb against a range of SARS-CoV-2 VOCs with and without TEV cleavage. IC_50_ values shown are the average of two independent experiments. The red dotted line indicates the average neutralization of Fc-ACE2 as in Supplementary Fig. 12. **d**, Intact IgG CrossMAb ReconnAbs (orange) show neutralization of SARS-CoV-2 BA.2. Inhibition is markedly reduced upon TEV cleavage (teal). The CrossMAb IgG ReconnAb neutralizes the BA.2 Omicron variant at ~50 pM NT_50_. Error bars denote standard deviation. A representative plot is shown.

We next investigated if ReconnAbs were able to neutralize lentiviral pseudoviruses corresponding to the SARS-CoV-2 VOCs and found that all ReconnAbs neutralized all VOCs, some showing nanomolar potency against Omicron (Fig. 4a). Consistent with its lower affinity (Fig. 3g), COV2-2143–ACE2 had the weakest neutralization of the tested ReconnAbs (Fig. 4a). COV2-2449–ACE2 showed the least deviation in neutralization potency among variants, consistent with its epitope being the most highly conserved (Extended Data Fig. 4b,c). Moreover, in live virus assays, CV10, CV27 and COV2-2449 showed neutralization against Wuhan-1 and Omicron SARS-CoV-2 virus (Supplementary Table 1). Neutralization is, as expected, slightly lower in this format given the use of a limiting dilution assay^40^. Notably, the TEV-proteolyzed versions of the ReconnAbs did not confer the same neutralizing potency as their uncleaved counterparts (Fig. 4a), demonstrating that the separate components are not working synergistically, but that the tether is essential for the ReconnAb components to work cooperatively. To examine if our selected linker length was sufficient to confer the desired activity, we investigated an additional longer linker length for two scFv–ACE2 fusions, from two distinct epitopes. Our CV27–ACE2 and COVA2-14–ACE2 fusions did not show any improved neutralization with an additional seven amino acids in the linker, suggesting that the examined linker length is sufficient to confer the desired activity (Extended Data Fig. 6b–d).

### Bi-functional, IgG-based ReconnAb development

Two ReconnAbs, CV10–ACE2 and COV2-2449–ACE2, were of particular interest as they showed broad-spectrum neutralization (Fig. 4a) and did not have overlapping epitopes (Fig. 2f). We postulated that a bi-functional IgG ReconnAb containing both CV10 and COV2-2449 would make viral escape even less likely, because it could bind to two distinct conserved epitopes. To produce a bi-functional IgG ReconnAb, we applied the clinically used^41^ CrossMAb platform^42^ and tethered ACE2 to the LC of only one of the IgG arms (Fig. 4b). This allows, as with the scFv–ACE2 fusions, a stoichiometry of only a single ACE2 per ReconnAb, such that ACE2 remains monovalent before and after TEV cleavage.

**Extended Data Fig. 7:**
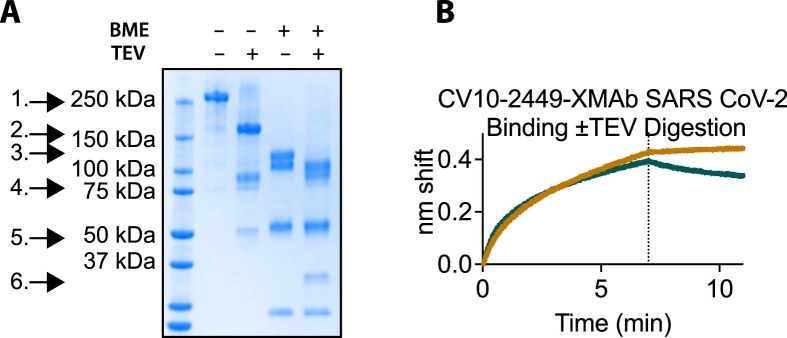
The bifunctional IgG-based ReconnAb expresses and binds to SARS-CoV-2 spike as expected. (A) The CV10-2449-ACE2-CrossMAb ReconnAb contains all designed components by SDS-PAGE, assayed before or after TEV cleavage and with or without 2-mercaptoethanol (BME). 1. Full-length ReconnAb, 2. Cleaved CrossMAb CV10-2449 IgG, 3. COV2-2449-LC-ACE2 fusion, 4. ACE2 (reduced ACE2 shows double banding), 5. HC, 6. Cleaved 2449-LC with linker, bottom band CV10 LC. (B) BLI binding of CV10-2449-ACE2-CrossMAb (brown) or the TEV cleaved form (teal) to SARS-CoV-2 spike. Binding is reduced upon TEV cleavage.

**Extended Data Fig. 8:**
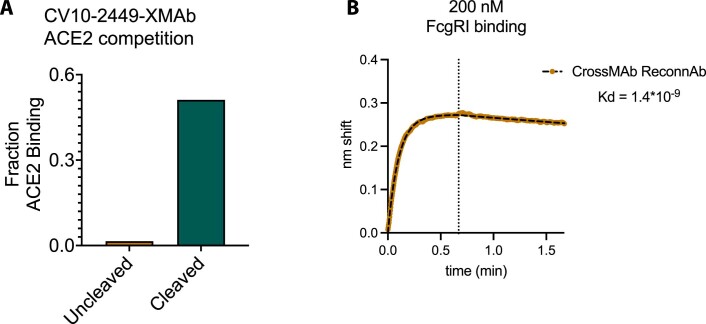
The bifunctional IgG-based ReconnAb blocks ACE2 binding and interacts with FcγRI. (A) Relative hFc-ACE2 binding to SARS-CoV-2 spike protein which has been pre-associated with 200 nM CV10-2449-XMAb (left) or the TEV cleaved form (right). No competitor was set to a value of 1.0. (B) The CrossMAb IgG ReconnAb binds to FcγRI, CrossMAb IgG was loaded on the octet using FAB2G tips and then associated with human FcγRI at 200 nM. The results demonstrate specific binding at an appropriate affinity.

**Extended Data Fig. 9:**
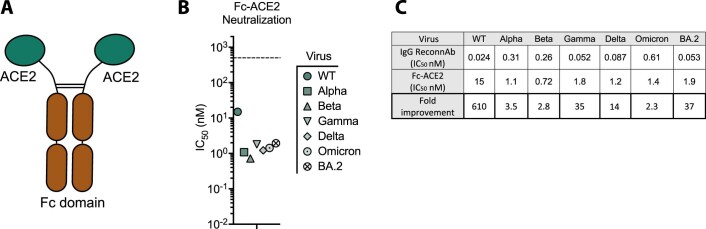
The CrossMAb IgG ReconnAb neutralizes better than bivalent ACE2 (Fc-ACE2). (A) Schematic depicting the protein design for Fc-tagged ACE2. Fc fusion was put C-terminal of the ACE2 ectodomain. (B) Neutralization of Fc-ACE2 against the same VOCs tested as in Fig.6 depicts weaker neutralization compared to the IgG ReconnAb. (C) a table of neutralizing potency of the IgG ReconnAb compared to Fc-ACE2 and the fold improvement for the IgG ReconnAb compared to Fc-ACE2.

We expressed and purified the CV10-2449–ACE2 CrossMAb (Extended Data Fig. 7a) and found that it bound to SARS-CoV-2 as expected (Extended Data Fig. 7b). As well, the uncleaved CrossMAb competed substantially with ACE2 (Extended Data Fig. 8a) and showed binding to FcγRI (Extended Data Fig. 8b). Finally, dependent on the presence of the tether, the CV10-2449–ACE2 CrossMAb neutralized all SARS-CoV-2 VOCs, including Omicron, at sub-nanomolar concentrations (Fig. 4c). Moreover, we found that our IgG ReconnAb was able to neutralize the BA.2 variant of Omicron (Fig. 4d). The neutralization potency generated by our IgG ReconnAb, with a monovalent ACE2, was substantially more robust than that generated by bivalent ACE2 (Fc-ACE2) alone (Extended Data Fig. 9), suggesting that the non-neutralizing binding component is conferring more benefit than an additional, low-affinity neutralizing component. These results were similarly reflected in a limiting dilution live viral neutralization assay (Supplementary Table 1). Taken together, the results described here lay a foundation for the development of ReconnAbs as a novel class of broadly neutralizing therapeutics.

## Discussion

Traditionally, the discovery of therapeutic biologics against infectious diseases has focused on identifying agents with neutralizing activity. We demonstrate here using ReconnAbs that cross-reactive, non-neutralizing antibodies, which have been often largely overlooked, can be powerful reagents in the creation of potent, broad-spectrum anti-viral agents.

ReconnAbs have two main components: a binding component, the non-neutralizing antibody that binds with high affinity to a conserved region on the spike protein, and an inhibitory component, in our case the ACE2 domain^43^. Because therapeutics containing ACE2 run the risk of eliciting autoimmunity in humans, our use of ACE2 as the inhibitory component represents a proof of concept of the ReconnAb design. The ACE2 module could be replaced by other neutralizing components, such as ACE2 domains with enhanced RBD-binding activity^44,45^, aptamers^46^ or RBD-directed mAbs^1,36–38,47^. It is noteworthy that we observed broad-spectrum efficacy with our ReconnAbs using monovalent ACE2, which, by itself, is weakly neutralizing; this suggests that, if a ReconnAb were made using a high-affinity RBD-directed antibody, efficacy would be sustained even if RBD escape mutations decreased affinity substantially. Future ReconnAb designs could also target the interaction with dipeptidyl peptidase 4 (DPP4), a receptor for other coronaviruses^48^, furthering their breadth.

Improvements could also be made to the conserved, non-neutralizing antibodies. Our library of SARS-CoV-2 non-RBD antibodies was derived from sequences early in the COVID-19 pandemic, which is relatively small in scope. The library does not contain, for instance, any vaccine-derived antibodies, which are known to include cross-reactive, non-neutralizing antibodies^25^. Future iterations of this work could start with much larger libraries^33^, with the potential to identify antibodies and/or nanobodies that target the most highly conserved epitopes and that are least likely to undergo mutational escape^49^. Although we have focused on non-neutralizing antibodies, neutralizing antibodies that bind to conserved epitopes might also be useful as the conserved component of ReconnAbs. Other features, such as linkage locations and length, fusion partners and modifications to the Fc domains, can be tuned in subsequent ReconnAb designs and will likely play an important role in their future conversion to therapeutics^50^.

Compared to neutralizing epitopes, highly conserved, non-neutralizing epitopes are less likely, in theory, to be subject to immune pressure, because antibody binding at these sites does not affect the ability of the virus to infect cells. Omicron provides strong evidence that SARS-CoV-2 viral evolution responds to immune pressure by mutating neutralizing epitopes (Fig. 1)^2,3,16^. ReconnAbs demonstrate the powerful utility of a non-active component, if it targets a highly conserved epitope, in the development of therapeutics. Indeed, we consider ReconnAbs to be a considerably more viable long-term option for SARS-CoV-2 therapy than the current standard where new monoclonals will need to be developed—for example, bebtelovimab^13^ for BA.2—with new emerging variants.

Finally, we anticipate that interrogation of existing antibody libraries for highly conserved, non-neutralizing binders will facilitate production of ReconnAbs, not just for SARS-CoV-2 but also for other viruses, such as HIV-1, influenza or other hCoVs. We see ReconnAbs as having utility not only in the current pandemic but also in mitigating the impact of future pandemics. Strategic stockpiles of customized ReconnAbs and rapid administration in a pandemic setting could alleviate the initial impact of a new pathogen, allowing time for other therapeutics and countermeasures to be put into place.

## Methods

### Determination of sequence conservation in SARS-CoV-2 spike

Sequences of 42 spike proteins, with between 30% and 90% sequence conservation compared to SARS-CoV-2, as well as RaTG13, were aligned using the 6VXX (Protein Data Bank (PDB)) sequence^51^ as a template. The sequences were aligned using Clustal Omega^52^ to develop a multiple sequence alignment (MSA). The MSA was uploaded onto the ConSurf server^53–55^ for overlay onto the 6VXX structure on chain A. The resultant chain A was re-colored based on conservation and replicated to replace chains B and C. The sequence alignment was again produced using MUSCLE^56^, and sequence identity was calculated using Geneious (Geneious Prime 2022.0.1). The alignment was truncated at residue 1,213 where the sequence alignment dropped to only nine sequences. Data were visualized using Pymol version 2.3.4. The sequences used were: 6VXX, _U5NJG5, _L7UP8, _A0A7U3W1C7, _K9N5Q8, _A0A2I6PIW5, _A0A3Q8AKM0, _U5WHZ7, _A0A5H2WTJ3, _A0A0U1WJY8, _A0A166ZND9, _A0A678TRJ7, _A0A2R4KP93, _A0A2Z4EVK1, _A0A7R6WCE7, _E0ZN36, _A0A6M3G9R1, _F1DAZ9, _A0A0U1UYX4, _A0A2R3SUW7, _A0A2Z4EVN5, _A0A2Z4EVN2, _U5LMM7, _A0A5Q0TVR4, _E0XIZ3, _A0A023Y9K3, _A0A2R4KP86, _A0A088DJY6, _A0A7G6UAJ9, _S4X276, _A0A4Y6GL90, _A3EXG6, _F1BYL9, _E0ZN60, _A0A0K1Z054 and _A0A0U1WHI2 and National Center of Biotechnology Information (NCBI) accession numbers YP_009047204.1, QLR06867.1, AAK32191.1, AGZ48828.1, AAT84362.1, QHR63300.2, ABD75513.1 and YP_003767.1.

### Library design

A library of antibodies directed against SARS-CoV-2 spike protein was developed using paired antibody sequences, meaning antibody sequences for which the HC and LC are both known, from the CoV-AbDab^23,33,36–38^. All antibody sequences from convalescent COVID-19 donors deposited before 9 July 2021 were inserted into a table and categorized by their binding to the SARS-CoV-2 RBD portion of the spike protein or to a non-RBD portion of SARS-CoV-2 spike. Antibodies cataloged for non-RBD binding were preferentially identified, resulting in a total of 385 paired antibody sequences. For these non-RBD-binding antibodies, the amino acid sequences of the corresponding HC and LC V-genes and CDR3 regions, already compiled from the CoV-AbDab, were imported into Geneious Prime version 2021.1.1 (https://www.geneious.com/). Using Geneious Prime, the HC sequences and LC sequences were separately analyzed to produce phylogenetic trees. For these phylogenetic trees, RBD-binding antibodies were also included to ensure selection of antibody sequences that were both non-RBD binding and clearly distinct from RBD-binding sequences. Three hundred seventy-one RBD-binding antibodies, 59 germline antibodies and 325 non-RBD-binding antibody nucleic acid sequences of the corresponding HC and LC genes were imported, for a total of 755 HC and LC sequences (696 excluding germline antibodies). The sequences were first aligned using the MUSCLE algorithm, and then two phylogenetic trees were made, both using PhyML 3.3.20180621. The sequence similarities used to produce phylogenetic trees account for antibody germlines, CDR lengths and amount of somatic hypermutation. After producing phylogenetic trees based on the HC and LC sequences, a total of 48 sequences were identified based on their location in the phylogeny. Distinct clusters, composed of only non-RBD sequences, on the HC phylogenetic trees were noted, and a single representative sequence was selected from each, chosen to also include distinct LC sequences whenever possible.

### scFv design

The sequences of these 48 antibodies were then converted into scFv sequences by linking the HC variable region to the LC variable region with a G4S-3 linker (GGGGSGGGGSGGGGS). All scFvs were designed in the following order: signal sequence-HC-G4S-3-LC. This vector also contained the HVM06_Mouse Ig HC V region 102 signal peptide (MGWSCIILFLVATATGVHS) to allow for protein secretion and purification from the supernatant. After construct design, the plasmids were ordered with the sequences inserted at the XhoI and NheI sites in the pTwist CMV BetaGlobin vector (Twist Biosciences).

### Library production

ScFvs were produced as Aga2p fusions^57^. In brief, 4 µg of pPNL6 vector in Cut Smart buffer was digested using 1 µl of NheI HF and BamHI HF (New England Biolabs) at 37 °C for 1 hour. Digested plasmid was then gel extracted using the Thermo Fisher Scientific Gel Extraction Kit. Equimolar aliquots of each scFv plasmid were pooled, and the resultant pool was amplified using primers that annealed to the hexa-his tag (reverse primer) or signal peptide (forward primer) and had a 50-bp overlap with the pPNL6 vector digested with NheI and BamHI. The pooled amplification was gel extracted to ensure that it was the correct size. Yeast were prepared by first streaking a YPAD plate and incubating for 2–3 days until single colonies were identifiable. A single colony was inoculated in 5 ml of YPAD with overnight shaking at 30 °C. Cultures were harvested into six tubes and pelleted. Yeast were resuspended in electroporation buffer (10 mM Tris base, 250 mM sucrose, 2 mM MgCl_2_) containing the gel-extracted library amplification and digested pPNL6 vector. This mixture was then pulsed, and the electroporated yeast were recovered in SD-CAA media overnight (30 °C shaking). These yeast were then induced by a 1:10 dilution into SG-CAA media and grown at 20 °C shaking for 2–3 days.

### Yeast binding

After induction in SG-CAA shaking for 2–3 days at 20 °C, the yeast library, expressing surface-exposed scFvs, was incubated for 15 minutes with a dilution of pre-formed baits. Baits were formed by mixing biotinylated baits and streptavidin 647 (Jackson ImmunoResearch) at a 4:1 ratio. For example, 250 nM bait would be produced by incubation of 250 nM biotinylated antigens and 62.5 nM streptavidin 647. Yeast were flowed with two colors of ‘bait’, the first (FITC) stains for a c-myc tag. The c-myc tag is a surrogate for expression as the scFv constructs in the pPNL6 vector contain an in-frame C-terminal c-myc tag. So, any yeast that are positive for c-myc are displaying full-length antibodies. The second color bait (Alexa Fluor 64 (APC channel)) stains for the antigen target of the scFv. To make the stain, streptavidin with an Alexa Fluor 647 tag is incubated with biotinylated bait protein. This complex is then used to stain the yeast. Any yeast that are positive for Alexa Fluor 647 are then binding to the protein antigen. Yeast were spun down and resuspended in 50 µl of PBSM containing the respective concentration of tetrameric bait. After 15 minutes, cells were then washed 1× with PBSM and resuspended in 50 µl of PBSM containing 1 µl of anti-c-myc FITC (Miltenyi Biotec) for 15 minutes. Samples were then washed 2× with PBSM and resuspended in 50 µl of PBSM. These samples were flowed (Accuri C6 flow cytometer, BD CSampler Plus software, version 1.0.34.1), and the percent antigen-positive was determined as the ratio of antigen-positive cells divided by all cells expressing scFv (c-myc-positive) multiplied by 100. Gates were set such that ~0.5% of yeast were antigen-positive in the streptavidin-alone control.

### Yeast sorts

The yeast library was incubated with 125 nM of tetrameric SARS-CoV-1 and 1 µl of anti-c-myc FITC (Miltenyi Biotec) for 1 hour. Samples were then washed 2× with PBSM and resuspended in 50 µl of PBSM. These libraries were then sorted on a FACSAria IIu using the Stanford FACS Facility. The samples were gated such that all antigen-positive cells were collected (gates set such that ~0.5% anti-c-myc FITC alone controls fell within the gate). Two populations were sorted: a hi-gate, consisting of the highest intensity binders (3.8% of all cells), and a low-gate, consisting of all other antigen-positive cells (3.7% of all cells). Cells were sorted directly into tubes containing 4 ml of SD-CAA media. These sorted libraries were grown for 1 day at 30 °C shaking in SD-CAA media, and then 300 µl of the cultures were mini-prepped (Zymo Research), following the manufacturer’s protocol. Mini-prepped DNA was transformed into STELLAR Competent Cells (Clontech) and plated on carbenicillin LB agar plates (as per pPNL6ʼs resistance marker). *Escherichia coli* cells that grow should, theoretically, contain only a single sequence from each of the yeast that were sorted above. Ten *E. coli* colonies from the hi-gate and 20 *E. coli* colonies from the low-gate sort were sent for sequencing (Sequetech). The sequences were then analyzed by sequence alignment using SnapGene software (version 6.0.2).

### Constructs

#### scFv–ACE2 fusion proteins

scFvs identified as cross-reacting with SARS-CoV-1 and falling into a unique epitope (CV10, CV27, COVA2-14, COV2-2449 and COV2-2143) sort were cloned into the pTwist CMV BetaGlobin vector such that they contained a linker (GGSGSHHHHHHASTGGGSGGPSGQAGAAASEENLYFQGSLFVSNHAYGGSGGEARV), followed by the ectodomain of human ACE2.

#### LC and LC–ACE2 fusion proteins

Antibody sequences were cloned into the CMV/R plasmid backbone for expression under a CMV promoter. The antibodies variable LC were cloned between the CMV promoter and the bGH poly(A) signal sequence of the CMV/R plasmid to facilitate improved protein expression. The variable region was cloned into the human IgG1 backbone with a kappa LC. This vector also contained the HVM06_Mouse (P01750) Ig HC V region 102 signal peptide to allow for protein secretion and purification from the supernatant. The LCs from the scFvs from the above-described SARS-CoV-1 sort were cloned into the CMV/R vector in-frame with the kappa LC. For COV2-2449, the LC was additionally cloned such that there was a C-terminal linker (GGSGSHHHHHHASTGGGSGGPSGQAGAAASEENLYFQGSLFVSNHAYGGSGGEARV), followed by the ectodomain of human ACE2.

### HC IgG plasmids

Antibody sequences were cloned into the CMV/R plasmid backbone for expression under a CMV promoter. The antibodies variable HC were cloned between the CMV promoter and the bGH poly(A) signal sequence of the CMV/R plasmid to facilitate improved protein expression. The variable region was cloned into the human IgG1 backbone. This vector also contained the HVM06_Mouse (P01750) Ig HC V region 102 signal peptide to allow for protein secretion and purification from the supernatant. The HCs from the scFvs from the above-described SARS-CoV-1 sort were cloned into the CMV/R vector in-frame with HC constant regions.

### hCoV spike protein constructs

Spike proteins from six hCoVs were cloned into a pADD2 vector between the rBeta-globin intron and β-globin poly(A). A total of 48 constructs were cloned and tested containing a C-terminal truncation or not, a T4 foldon or GCN4 trimerization domain and an Avi tag or not. Each set of eight proteins was produced for the six hCoV spike proteins from SARS-CoV-2, SARS-CoV-1, MERS, 229E, NL63 and OC43. Depictions of the constructs and linkers produced are shown in Extended Data Fig. 2.

### Lentivirus plasmids

Plasmids encoding the full-length spike proteins with native signal peptides were cloned into the background of the HDM-SARS2-spike-delta21 plasmid (Addgene plasmid, 155130). This construct contains a 21-amino acid C-terminal deletion to promote viral expression. The SARS-CoV-1 spike was used with an 18-amino acid C-terminal deletion. The other viral plasmids that were used were previously described^58^. They include pHAGE-Luc2-IRS-ZsGreen (NR-52516), HDM-Hgpm2 (NR-52517), pRC-CMV-Rev1b (NR-52519) and HDM-tat1b (NR-52518).

### DNA preps

The 48 spike protein constructs from the hCoVs were mirA-prepped^59^ using Thermo Fisher Scientific GeneJET plasmid mini-prep kit. Eight milliliters of *E. coli* containing the constructs were harvested by centrifugation, and 200 µl of freshly made resuspension buffer was added to each clone. Then, 200 µl of lysis buffer was added, followed by inversion, and then 300 µl of neutralization buffer was added. Lysed *E. coli* was then centrifuged at >18,000*g* for 10 minutes. The supernatant was transferred to a tube containing 580 µl of 100% EtOH. The EtOH solution was then added to a GeneJET plasmid mini-prep column, and the regular wash steps and elution steps were followed. mirA-preps resulted in significantly more plasmid production and allowed for small-scale transfection of the 48 clones tested. For the FL-GCN4-Avi-His expression tests and protein production, all samples were maxi-prepped from 200 ml of *E. coli* using NuceloBond Xtra Maxi Kit per the manufacturer’s recommendations (Macherey-Nagel). All scFv-ACE2, CrossMAb^60^, antibody, hFc-ACE2 and lentiviral plasmids were maxi-prepped in the same fashion.

### Protein production

All proteins were expressed in Expi293F cells. Expi293F cells were cultured in media containing 66% Freestyle/33% Expi media (Thermo Fisher Scientific) and grown in TriForest polycarbonate shaking flasks at 37 °C in 8% CO_2_. The day before transfection, cells were spun down and resuspended to a density of 3 × 10^6^ cells per ml in fresh media. The next day, cells were diluted and transfected at a density of approximately 3–4 × 10^6^ cells per ml. Transfection mixtures were made by adding the following components: mirA-prepped or maxi-prepped DNA, culture media and FectoPro (Polyplus) would be added to cells to a ratio of 0.5–0.8 µg:100 µl:1.3 µl:900 µl. For example, for a 100-ml transfection, 50–80 µg of DNA would be added to 10 ml of culture media, and then 130 µl of FectoPro would be added to this. After mixing and a 10-minute incubation, the resultant transfection cocktail would be added to 90 ml of cells. The cells were harvested 3–5 days after transfection by spinning the cultures at >7,000*g* for 15 minutes. Supernatants were filtered using a 0.22-µm filter. To determine hCoV protein expression, spun-down Expi293F supernatant was used without further purification. For proteins containing a biotinylation tag (Avi-Tag), Expi293F cells containing a stable BirA enzyme insertion were used, resulting in spontaneous biotinylation during protein expression.

### Protein purification—Fc Tag-containing proteins

All proteins containing an Fc tag (for example, IgGs, CrossMAb–Ace2 fusions and hFc-ACE2) were purified using a 5-ml MAb Select SuRe PRISM column on the ÄKTA pure fast protein liquid chromatography (FPLC) system (Cytiva). Filtered cell supernatants were diluted with 1/10 volume of 10× PBS. The ÄKTA system was equilibrated with: A1 – 1× PBS; A2 – 100 mM glycine pH 2.8; B1 – 0.5 M NaOH; Buffer line – 1× PBS; and Sample lines – H_2_O. The protocol washes the column with A1, followed by loading of the sample in Sample line 1 until air is detected in the air sensor of the sample pumps, followed by 5 column volume washes with A1 and elution of the sample by flowing of 20 ml of A2 (directly into a 50-ml conical containing 2 ml of 1 M Tris pH 8.0), followed by 5 column volumes of A1, B1 and A1. The resultant Fc-containing samples were concentrated using 50-kDa or 100-kDa cutoff centrifugal concentrators. Proteins were buffer exchanged using a PD-10 column (Sephadex) that had been pre-equilibrated into 20 mM HEPES and 150 mM NaCl. IgGs used for competition, binding and neutralization experiments were not further purified. CrossMAb–ACE2 fusions were then further purified using the S6 column on the ÄKTA system.

### Protein purification—His-tagged proteins

All proteins not containing an Fc tag (for example, scFvs and scFv fusions and FL spike trimers from hCoV polypeptide antigens) were purified using HisPur Ni-NTA resin (Thermo Fisher Scientific). Cell supernatants were diluted with 1/3 volume of wash buffer (20 mM imidazole, 20 mM HEPES pH 7.4 and 150 mM NaCl), and the Ni-NTA resin was added to diluted cell supernatants. For all mixtures not containing hCoV spike protein, the samples were then incubated at 4 °C while stirring overnight. hCoV spike proteins were incubated at room temperature. Resin–supernatant mixtures were added to chromatography columns for gravity flow purification. The resin in the column was washed with wash buffer (20 mM imidazole, 20 mM HEPES pH 7.4 and 150 mM NaCl), and the proteins were eluted with 250 mM imidazole, 20 mM HEPES pH 7.4 and 150 mM NaCl. Column elutions were concentrated using centrifugal concentrators (50-kDa cutoff for scFv–ACE2 fusions and 100-kDa cutoff for trimer constructs), followed by size-exclusion chromatography on an ÄKTA pure system. ÄKTA pure FPLC with a Superdex 6 Increase gel filtration column (S6) was used for purification. Then, 1 ml of sample was injected using a 2-ml loop and run over the S6, which had been pre-equilibrated in de-gassed 20 mM HEPES and 150 mM NaCl before use. Biotinylated antigens were not purified using the ÄKTA pure system.

### TEV digestion

TEV digestion of scFv–ACE2 fusions. Two microliters of TEV protease (New England Biolabs) was added per 200 µl of scFv–ACE2 fusions at ~4 µM in 20 mM HEPES and 150 mM NaCl. The reaction was left to incubate overnight at 30 °C. Extent of cleavage was determined by SDS-PAGE analysis on 4–20% Mini-PROTEAN TGX protein gels stained with GelCode Blue Stain Reagent (Thermo Fisher Scientific). For, TEV digestion of CrossMAb–ACE2 fusions, 3 µl of TEV protease (New England Biolabs) was added per 200 µl of CrossMAb–ACE2 fusions at ~2 µM in 20 mM HEPES and 150 mM NaCl. The reaction was left to incubate overnight at 30 °C. Extent of cleavage was determined by SDS-PAGE analysis on 4–20% Mini-PROTEAN TGX protein gels stained with GelCode Blue Stain Reagent (Thermo Fisher Scientific).

### Fab production from IgGs

1/10 volume of 1 M Tris pH 8 was added to IgGs at ~2 mg ml^−1^ in PBS. Then, 2 µl of a 1 mg ml^−1^ stock of Lys-C (stock stored at −70 °C) was added for each mg of human IgG1 and digested for 1 hour at 37 °C with moderate rotation. Digested Fabs were purified by SP/ÄKTA using 50 mM NaOAc pH 5 with gradient NaCl elution (using 50 mM NaOAc + 1 M NaCl pH 5). Fab fractions were pooled and dialyzed against 1× PBS and concentrated using 30-kDa concentrators. Purified Fabs were stored at −80 °C.

### BLI binding

BLI (Octet) binding experiments—hCoV expression testing. All reactions were run on an Octet RED96, and samples were run in PBS with 0.1% BSA and 0.05% Tween 20 (Octet buffer). hCoV supernatants were assessed for binding using Anti-Penta His (His1K) tips. These tips are designed to bind specifically to a Penta-His tag on proteins. For this experiment, tips were baselined in a blank well and then associated in the wells containing 50 µl of hCoV expression media and 150 µl of Octet buffer. Response values (that is, peak reached after 5 minutes of association) were determined using the Octet data analysis software. Final data analysis was done in Prism.

BLI (Octet) binding experiments—IgG binding. All reactions were run on an Octet RED96, and samples were run in PBS with 0.1% BSA and 0.05% Tween 20 (Octet buffer). IgGs produced from the scFvs from the above sort were assessed for binding using streptavidin biosensors (Sartorius/ForteBio) loaded to a threshold of 0.8 nm of SARS-CoV-2, SARS-CoV-1, MERS and OC43 biotinylated spike proteins. Tips were then washed and baselined in wells containing only Octet buffer. Samples were then associated in wells containing 100 nM IgG. A control well that loaded antigen but associated in a well containing only 200 µl of Octet buffer was used as a baseline subtraction for data analysis.

BLI (Octet) binding experiments—IgG competition. All reactions were run on an Octet RED96, and samples were run in PBS with 0.1% BSA and 0.05% Tween 20 (Octet buffer). IgGs produced from the scFvs from the above sort were assessed for their competition of binding with one another using Anti-Penta His (His1K) biosensors (Sartorius/ForteBio). His1K tips were pre-quenched with buffer containing 10 nM biotin. Tips were then loaded with 100 nM protein for 2 minutes (SARS-CoV-2 spike) or 4 minutes (SARS-CoV-1 spike). These tips were then associated with one of seven antibodies (CV27, COV2-2147, CV10, COVA2-14, COVA2-18, COV2-2449 or COV2-2143) at 100 nM for 5 minutes to reach saturation. Tips were baselined and then associated with one of the seven antibodies. For this step, all eight tips went into the same antibody at 100 nM. Response values (that is, peak reached after 2 minutes of association) was determined using Octet data analysis software. Values were normalized to the tip loaded with either SARS-CoV-2 or SARS-CoV-1 spike but without a competing antibody. These values were set as a value of 1 for each antibody. This is simply the antibody binding to the protein. Additionally, the antibody competing with itself was set to a value of 0. Final data analysis was done in Prism.

BLI (Octet) binding experiments—scFv–ACE2 fusion. All reactions were run on an Octet RED96, and samples were run in PBS with 0.1% BSA and 0.05% Tween 20. Streptavidin biosensors (Sartorius/ForteBio) were loaded for 2 minutes with 100 nM biotinylated antigens (SARS-CoV-2 or SARS-CoV-1 spike proteins). Samples were then washed and baselined in wells containing Octet buffer. Association occurred in samples containing ACE2 fusion proteins either without or with TEV protease (New England Biolabs) treatment. scFv–ACE2 fusions were tested at 200 nM. Association was conducted for 2 minutes, and dissociation was conducted for 1 minute.

BLI (Octet) binding experiments—scFv–ACE2 fusion and CrossMAb–ACE2 fusion competition with hFc-ACE2 (ref. ^61^). All reactions were run on an Octet RED96, and samples were run in PBS with 0.1% BSA and 0.05% Tween 20 (Octet buffer). Streptavidin biosensors (Sartorius/ForteBio) (scFvs) or His1K biosensors (Sartorius/ForteBio) (CrossMAb) were loaded for 2 minutes with 100 nM biotinylated antigens (SARS-CoV-2 or SARS-CoV-1 spike—scFvs) or 4 minutes with 200 nM His-tagged antigens (CrossMAb). Samples were then washed and baselined in wells containing Octet buffer. scFv–ACE2 fusions or CrossMAbs were then associated for 5 minutes. Samples were baselined and then associated with either hFc-ACE2 for 2 minutes (scFv) or 40 seconds (CrossMAb). Response values was determined using Octet data analysis software. Samples that loaded SARS-CoV-2 or SARS-CoV-1 but did not associate with any hFc-ACE2 were used as a baseline subtraction. Values were normalized to the binding of hFc-ACE2 without a competitor.

### Lentivirus production

SARS-CoV-2, VOCs and SARS-CoV-1 spike pseudotyped lentiviral particles were produced. Viral transfections were done in HEK293T cells using calcium phosphate transfection reagent. Six million cells were seeded in D10 media (DMEM + additives: 10% FBS, L-glutamate, penicillin, streptomycin and 10 mM HEPES) in 10-cm plates 1 day before transfection. A five-plasmid system (plasmids described above) was used for viral production, as described in Crawford et al.^58^. The spike vector contained the 21-amino acid truncated form of the SARS-CoV-2 spike sequence from the Wuhan-Hu-1 strain of SARS-CoV-2 or VOCs or 18-amino acid truncation for SARS-CoV-1. VOCs were based off wild-type (WT) – sequence ID: BCN86353.1; Alpha – sequence ID: QXN08428.1; Beta – sequence ID: QUT64557.1; Gamma – sequence ID: QTN71704.1; Delta – sequence ID: QWS06686.1, which also has V70F and A222V mutations; and Omicron – sequence ID: UFO69279.1. The plasmids were added to D10 medium in the following ratios: 10 µg pHAGE-Luc2-IRS-ZsGreen, 3.4 µg FL spike, 2.2 µg HDM-Hgpm2, 2.2 µg HDM-Tat1b and 2.2 µg pRC-CMV-Rev1b in a final volume of 1,000 µl. To form transfection complexes, 30 µl of BioT (BioLand) was added. Transfection reactions were incubated for 10 minutes at room temperature, and then 9 ml of medium was added slowly. The resultant 10 ml was added to plated HEK cells from which the medium had been removed. Culture medium was removed 24 hours after transfection and replaced with fresh D10 medium. Viral supernatants were harvested 72 hours after transfection by spinning at 300*g* for 5 minutes, followed by filtering through a 0.45-µm filter. Viral stocks were aliquoted and stored at −80 °C until further use.

### Neutralization

The target cells used for infection in viral neutralization assays were from a HeLa cell line stably overexpressing the SARS-CoV-2 receptor, ACE2, as well as the protease known to process SARS-CoV-2, TMPRSS2. Production of this cell line is described in detail in ref. ^62^, with the addition of stable TMPRSS2 incorporation. ACE2/TMPRSS2/HeLa cells were plated 1 day before infection at 5,000 cells per well. Ninety-six-well white-walled, white-bottom plates were used for the assay (Thermo Fisher Scientific). On the day of the assay, purified CrossMAb–ACE2 or scFv–ACE2 fusions in HEPES (20 mM) and NaCl (150 mM), which either had or had not been treated with TEV protease (as above), were sterile filtered using a 0.22-µm filter. Dilutions of this filtered stock were made into sterile 1× DPBS (Thermo Fisher Scientific), which was 5% by volume D10 medium. Each dilution well contained 30 µl of CrossMAb–ACE2 or scFv–ACE2 fusions. Samples were run in technical duplicate in each experiment. Virus-only wells and cell-only wells contained only 30 µl of 1× DPBS.

A virus mixture was made containing the virus of interest (for example, SARS-CoV-2) and D10 media (DMEM + additives: 10% FBS, L-glutamate, penicillin, streptomycin and 10 mM HEPES). Virus dilutions into media were selected such that a suitable signal would be obtained in the virus-only wells. A suitable signal was selected such that the virus-only wells would achieve a luminescence of at least >10,000 RLU. Then, 90 µl of this virus mixture was added to each of the inhibitor dilutions to make a final volume of 120 µl in each well. Virus-only wells were made that contained 30 µl of 1× DPBS and 90 µl of virus mixture. Cell-only wells were made that contained 30 µl of 1× DPBS and 90 µl of D10 media.

The inhibitor/virus mixture was left to incubate for 1 hour at 37 °C. After incubation, the medium was removed from the cells on the plates made 1 day prior. This was replaced with 100 µl of inhibitor/virus dilutions and incubated at 37 °C for approximately 24 hours. At 24 hours after infection, the media was exchanged for fresh media in all samples containing a TEV-cleavable linker with our without cleavage; media was not exchanged on samples that did not have a TEV-cleavable linker (for example, WT IgGs). Infectivity readout was performed by measuring luciferase levels. Forty-eight hours after infection, 50 µl of medium was removed from all wells and cells were lysed by the addition of 50 µl of BriteLite assay readout solution (Perkin Elmer) into each well, alternatively, all the media was removed from the wells and 100 µL of a 1:1 dilution of BriteLite was used. Luminescence values were measured using a BioTek Synergy HT Microplate Reader (BioTek). Each plate was normalized by averaging cell-only (0% infectivity) and virus-only (100% infectivity) wells. Cell-only and virus-only wells were averaged. Normalized values were fit with a four-parameter non-linear regression inhibitor curve in Prism to obtain 50% inhibitory concentration (IC_50_) values. The average half-maximal neutralization titer (NT_50_) of two independent experiments are shown.

### ELISA

IgG ELISAs against hCoV strains were performed. Streptavidin solution (5 µg ml^−1^) was plated in 50 µl in each well on a MaxiSorp (Thermo Fisher Scientific) microtiter plate in 50 mM sodium bicarbonate pH 8.75. This was left to incubate for 1 hour at room temperature. These were washed 3× with 300 µl of ddH_2_O using an ELx 405 Bio-Tex plate washer and blocked with 150 ul of ChonBlock (Chondrex) for at least 1 hour at room temperature. Biotinylated hCoV spike proteins were added to each well at a concentration of 1 µg ml^−1^ and left to incubate overnight at 4 °C. Plates were washed 3× with 300 µl of 1× PBST, and serial dilutions of monoclonal antibodies (described above) were added, starting at 1 µM and undergoing ten-fold serial dilutions. These were left to incubate for 1 hour at room temperature and then washed 3× with PBST. Goat anti-human HRP (Abcam, ab7153) was added at a 1:5,000 dilution in PBST. This was left to incubate at room temperature for 1 hour and then washed 6× with PBST. Finally, the plate was developed using 50 µl of 1-StepTM Turbo-TMB-ELISA Substrate Solution (Thermo Fisher Scientific) per well, and the plates were quenched with 50 µl of 2 M H_2_SO_4_ to each well. Plates were read at 450 nm and normalized for path length using a BioTek Synergy HT Microplate Reader.

### Dot blot analysis of hCoV expression

Expi293F culture supernatants from hCoV spike antigen expressions using mirA-preps as above were harvested 2 days after transfection via centrifugation at 7,000*g* for 15 minutes. Supernatants were spotted on a nitrocellulose membrane. The blot was dried for 15 minutes in a fume hood. After drying, 10 ml of 1× PBST + 5% blotting grade blocker (Bio-Rad) was added for 10 minutes. Two microliters of mouse anti-hexa His antibody (BioLegend) was added to the 10-ml sample (final 1:5,000) and incubated for 1 hour at room temperature. Blots were washed 16× with 9 ml of PBST. Ten milliliters of 1× PBST + 5% blotting grade blocker with 2 μl of anti-mouse IgG1 (Abcam, final 1:5,000) were added and incubated for 1 hour at room temperature. Blots were washed 16× with 9 ml of PBST, developed using Pierce ECL western blotting substrate and imaged using a GE Amersham Imager 600.

### Live SARS-CoV-2 virus isolation and passages

Variants were obtained from two sources. WA-1/2020 was obtained from WRCEVA. BA.1 and BA.2 were isolated from de-identified nasopharyngeal (NP) swabs sent to the California Department of Public Health from hospitals in California for surveillance purposes. To isolate from patient swabs, 200 µl of an NP swab sample from a patient with COVID-19 that was previously sequence-identified was diluted 1:3 in PBS supplemented with 0.75% BSA (BSA-PBS) and added to confluent Vero E6-TMPRSS2-T2A-ACE2 cells in a T25 flask, allowed to adsorb for 1 hour, inoculum removed, and additional media was added. The flask was incubated at 37 °C with 5% CO_2_ for 3–4 days with daily monitoring for cytopathic effects (CPE). When 50% CPE was detected, the contents were collected, clarified by centrifugation and stored at −80 °C as passage 0 stock. Passaged stock was made by inoculation of Vero E6-TMPRSS2-T2A-ACE2 confluent T150 flasks with 1:10 diluted passage 0 stock, similarly monitored and harvested at approximately 80% CPE. All viral stocks were sequenced to confirm lineage, and 50% tissue culture infectious dose (TCID_50_) was determined by titration.

### Live SARS-CoV-2 virus 50% CPE endpoint neutralization

CPE endpoint neutralization assays were done following the limiting dilution model using sequence-verified viral stocks of WA-1, BA.1 and BA.2 in Vero E6-TMPRSS2-T2A-ACE2. Three-fold serial dilutions of inhibitor were made in BSA-PBS and mixed at a 1:1 ratio with 100 TCID_50_ of each virus and incubated for 1 hour at 37 °C. Final inhibitor dilutions ranged from 500 nM to 0.223 nM. Then, 100 µl of the plasma/virus mixtures were added in duplicate to flat-bottom 96-well plates seeded with Vero E6-TMPRSS2-T2A-ACE2 at a density of 2.5 × 10^4^ per well and incubated in a 37 °C incubator with 5% CO_2_ until consistent CPE was seen in the virus control (no inhibitor added) wells. Positive and negative controls were included as well as cell control wells and a viral back titration to verify TCID_50_ viral input. Individual wells were scored for CPE as having a binary outcome of ‘infection’ or ‘no infection’, and the ID_50_ was calculated using the Spearman–Karber method. All steps were done in a Biosafety Level 3 laboratory using approved protocols.

### Reporting summary

Further information on research design is available in the Nature Research Reporting Summary linked to this article.

## Online content

Any methods, additional references, Nature Research reporting summaries, source data, extended data, supplementary information, acknowledgements, peer review information; details of author contributions and competing interests; and statements of data and code availability are available at 10.1038/s41589-022-01140-1.

## Supplementary information

Supplementary Information Supplementary Note, containing sequences for the proteins used in the study, and Supplementary Table 1 and legend.

Reporting Summary

## Data Availability

Antibody sequences were obtained from the CoV-AbDab and coronavirus spike protein alignment sequences from UniRef90. All antibody sequences examined, alignments and phylogenetic trees used are available on Dryad at 10.7272/Q68S4N53. Raw data are plotted as shown or included as tables.
