# Supplementary material for: Converting non-neutralizing SARS-CoV-2 antibodies into broad-spectrum inhibitors
Source: Nat Chem Biol. 2022 Sep 8;18(11):1270–6. doi: 10.1038/s41589-022-01140-1 (PMC9596371; doi:10.1038/s41589-022-01140-1)
Supplement: Supplementary file 1 — Supplementary Note, containing sequences for the proteins used in the study, and Supplementary Table 1 and legend. [file 41589_2022_1140_MOESM1_ESM.pdf]

nature portfolio

https://doi.org/10.1038/s41589-022-01140-1

Supplementary information

Converting non-neutralizing SARS-CoV-2 antibodies into broad-spectrum inhibitors

In the format provided by the authors and unedited

Supplementary Information:

Converting non-neutralizing SARS-CoV-2 antibodies into broad-spectrum inhibitors

Payton A.-B. Weidenbacher[1,2], Eric Waltari[3], Izumi de los Rios Kobara[4], Benjamin N. Bell[1,5], Mary Kate Morris[6], Ya-Chen Cheng[1,7], Carl Hanson[6], John E. Pak[3], Peter S. Kim[1,3,7]

[1] Stanford ChEM-H, Stanford University, Stanford, CA 94305, [2] Department of Chemistry, Stanford University, Stanford, CA 94305, [3] Chan Zuckerberg Biohub, San Francisco, CA 94158, [4] Stanford Immunology Program, Stanford University School of Medicine, Stanford, CA 94305, [5] Department of Molecular and Cellular Physiology, Stanford University School of Medicine, Stanford, California 94305, [6] California Department of Public Health, Richmond, California, USA, [7] Department of Biochemistry, School of Medicine, Stanford University, Stanford, CA 94305

Supplementary note:

Signal Peptide

Hexa-His tag

TEV Site

ACE2

CV10-ACE2 Fusion scFv

MGWSCIILFLVATATGVHSQVQLQESGPGLVKPSETLSLTCNVSGGSISSYYWSWIRQP
PGKGLEWIGYIYYSGSTNYNPSLKSRVTISVDTSKNQFSLKLSSVTAADTAVYYCARGFD
YWGQGTLVTVSSASGGGGSGGGGSGGGGSEIVLTQSPGTLSLSPGERATLSCRASQSVS

SIYLAWYQQKPGQAPRLLIYGASSRATGIPDRFSGSGSGTDFTLTISRLEPEDFAVYYCQQYAGSPWTFGQGTKVEIKGGSGS*HHHHHH*ASTGGGSGGPSGQAGAAASE**ENLYFQG**SLFVSNHAYGGSGGEARVSTIEEQAKTFLDKFNHEAEDLFYQSSLASWNYNTNITEENVQNMNNAGDKWSAFLKEQSTLAQMYPLQEIQNLTVKLQLQALQQNGSSVLSEDKSKRLNTILNTMSTIYSTGKVCNPDNPQECLLLEPGLNEIMANSLDYNERLWAWESWRSEVGKQLRPLYEEYVVLKNEMARANHYEDYGDYWRGDYEVNGVDGYDYSRGQLIEDVEHTFEEIKPLYEHLHAYVRAKLMNAYPSYISPIGCLPAHLLGDMWGRFWTNLYSLTVPFGQKPNIDVTDAMVDQAWDAQRIFKEAEKFFVSVGLPNMTQGFWENSMLTDPGNVQKAVCHPTAWDLGKGDFRILMCTKVTMDDFLTAHHEMGHIQYDMAYAAQPFLLRNGANEGFHEAVGEIMSLSAATPKHLKSIGLLSPDFQEDNETEINFLLKQALTIVGTLPFTYMLEKWRWMVFKGEIPKDQWMKKWWEMKREIVGVVEPVPHDETYCDPASLFHVSNDYSFIRYYTRTLYQFQFQEALCQAAKHEGPLHKCDISNSTEAGQKLFNMLRLGKSEPWTLALENVVGAKNMNVRPLLNYFEPLFTWLKDQNKNSFVGWSTDWSPYAD

CV27-ACE2 Fusion scFv

MGWSCIILFLVATATGVHSQVQLVESGGGVVQPGRSLRLSCAASGFTFSSYAMHWVRQAPGKGLEWVALISYDGSNKYYADSVKGRFTISRDNSKNTLYLQMNSLRAEDTAVYYCARSFGGSYYYGMDVWGQGTTVTASGGGGSGGGGSGGGGSQSALTQPASVSGSPGQSITISCTGTSSDVGGYNYVSWYQQHPGKAPKLMIYDVSNRPSGVSNRFSGSKSGNTASLTISGLQAEDEADYYCSSYTSSSTPYVFGTGTKVGGSGS*HHHHHH*ASTGGGSGGPSGQAGAAASE**ENLYFQG**SLFVSNHAYGGSGGEARVSTIEEQAKTFLDKFNHEAEDLFYQSSLASWNYNTNITEENVQNMNNAGDKWSAFLKEQSTLAQMYPLQEIQNLTVKLQLQALQQNGSSV

LSEDKSKRLNTILNTMSTIYSTGKVCNPDNPQECLLLEPGLNEIMANSLDYNERLWAWESWRSEVGKQLRPLYEEYVVLKNEMARANHYEDYGDYWRGDYEVNGVDGYDYSRGQLIEDVEHTFEEIKPLYEHLHAYVRAKLMNAYPSYISPIGCLPAHLLGDMWGRFWTNLYSLTVPFGQKPNIDVTDAMVDQAWDAQRIFKEAEKFFVSVGLPNMTQGFWENSMLTDPGNVQKAVCHPTAWDLGKGDFRILMCTKVTMDDFLTAHHEMGHIQYDMAYAAQPFLLRNGANEGFHEAVGEIMSLSAATPKHLKSIGLLSPDFQEDNETEINFLLKQALTIVGTLPFTYMLEKWRWMVFKGEIPKDQWMKKWWEMKREIVGVVEPVPHDETYCDPASLFHVSNDYSFIRYYTRTLYQFQFQEALCQAAKHEGPLHKCDISNSTEAGQKLFNMLRLGKSEPWTLALENVVGAKNMNVRPLLNYFEPLFTWLKDQNKNSFVGWSTDWSPYAD

COVA2-14-ACE2 Fusion scFv

MGWSCIILFLVATATGVHSQVQLVQSGAEVKKPGSSVKVSCKASGGTFSSYAIIWVRQAPGQGLEWMGGIIPIFGTANYAQKFQGRVTITTDESTSTAYMELSSLRSEDTAVYYCARVRYYDSSGYYEDYWGQGTLVTVSSASGGGGSGGGGSGGGGSEIVLTQSPATLSLSPGERATLSCRASQSVSSYLAWYQQEPGQAPRLLIYDASNRATGIPARFSGSGSGTDFTLTISSLEPEDFAVYYCQQRSNWPPMYTFGQGTKVEIKGGSGS*HHHHHH*ASTGGGSGGPSGQAGAAASE**ENLYFQG**SLFVSNHAYGGSGGEARVSTIEEQAKTFLDKFNHEAEDLFYQSSLASWNYNTNITEENVQNMNNAGDKWSAFLKEQSTLAQMYPLQEIQNLTVKLQLQALQQNGSSVLSEDKSKRLNTILNTMSTIYSTGKVCNPDNPQECLLLEPGLNEIMANSLDYNERLWAWESWRSEVGKQLRPLYEEYVVLKNEMARANHYEDYGDYWRGDYEVNGVDGYDYSRGQLIEDVEHTFEEIKPLYEHLHAYVRAKLMNAYPSYISPIGCLPAHLLGDMWGRFWTNLYSLTVPFGQKPNIDVTDAMVDQAWDAQRIFKEAEKFFVSVGLPNMTQGFWENSMLTDPG

NVQKAVCHPTAWDLGKGDFRILMCTKVTMDDFLTAHHEMGHIQYDMAYAAQPFLLR
NGANEGFHEAVGEIMSLSAATPKHLKSIGLLSPDFQEDNETEINFLLKQALTIVGTLPFTY
MLEKWRWMVFKGEIPKDQWMKKWWEMKREIVGVVEPVPHDETYCDPASLFHVSNDY
SFIRYYTRTLYQFQFQEALCQAAKHEGPLHKCDISNSTEAGQKLFNMLRLGKSEPWTLA
LENVVGAKNMNVRPLLNYFEPLFTWLKDQNKNSFVGWSTDWSPYAD

COV2-2449-ACE2 Fusion scFv

MGWSCIILFLVATATGVHSQVQLVESGGGVVQPGRSLRLSCATSGFTFSSFALHWVRQ
APGKGLEWVTVISDDGNNKYYVDSVKGRFTISRDNSKNTLFLQMNSLRVEDTAIYYCA
RASYNSNWSIGEYFRDWGQGTLVTVSSASGGGGSGGGGSGGGGSDIVMTQSPDSLAVS
LGERATINCKSSQSLLYTSNNKNYLAWYQQKPGQPPKLLIYWASTRESGVPDRFSGSGS
GTDFTLTISSLQAEDVAVYYCQQYYSPPWTFGQGTKVEIKGGSGS*HHHHHH*ASTGGGS
GGPSGQAGAAASE**ENLYFQG**SLFVSNHAYGGSGGEARVSTIEEQAKTFLDKFNHEAED
LFYQSSLASWNYNTNITEENVQNMNNAGDKWSAFLKEQSTLAQMYPLQEIQNLTVKLQ
LQALQQNGSSVLSEDKSKRLNTILNTMSTIYSTGKVCNPDNPQECLLLEPGLNEIMANSL
DYNERLWAWESWRSEVGKQLRPLYEEYVVLKNEMARANHYEDYGDYWRGDYEVNG
VDGYDYSRGQLIEDVEHTFEEIKPLYEHLHAYVRAKLMNAYPSYISPIGCLPAHLLGDM
WGRFWTNLYSLTVPFGQKPNIDVTDAMVDQAWDAQRIFKEAEKFFVSVGLPNMTQGF
WENSMLTDPGNVQKAVCHPTAWDLGKGDFRILMCTKVTMDDFLTAHHEMGHIQYDM
AYAAQPFLLRNGANEGFHEAVGEIMSLSAATPKHLKSIGLLSPDFQEDNETEINFLLKQA
LTIVGTLPFTYMLEKWRWMVFKGEIPKDQWMKKWWEMKREIVGVVEPVPHDETYCDP

ASLFHVSNDYSFIRYYTRTLYQFQFQEALCQAAKHEGPLHKCDISNSTEAGQKLFNMLRLGKSEPWTLALENVVGAKNMNVRPLLNYFEPLFTWLKDQNKNSFVGWSTDWSPYAD

COV2-2143-ACE2 Fusion scFv

MGWSCIILFLVATATGVHSEVQLVESGGGLVQPGGSLRLSCAASGFTVSSNYMSWVRQAPGKGLEWVSVIYSAGSTYYADSVKGRFSISRDKSKNTLYLQMNSLRAEDTAVYYCAKEGGSGSLRYYYYGMDVWGQGTTVTVSSASGGGGSGGGGSGGGGSQSVVTQPPSASGTPGQRVTISCSGSSSNIGYNIVNWYQQLPGTAPKLLIYSNNQRPSGVPDRFSGSKSGTSASLSISGLQSEDEADYYCAAWDDSLNGYVFGTGTKVTVLGGSGS*HHHHHH*ASTGGGSGGPSGQAGAAASE**ENLYFQG**SLFVSNHAYGGSGGEARVSTIEEQAKTFLDKFNHEAEDLFYQSSLASWNYNTNITEENVQNMNNAGDKWSAFLKEQSTLAQMYPLQEIQNLTVKLQLQALQQNGSSVLSEDKSKRLNTILNTMSTIYSTGKVCNPDNPQECLLLEPGLNEIMANSLDYNERLWAWESWRSEVGKQLRPLYEEYVVLKNEMARANHYEDYGDYWRGDYEVNGVDGYDYSRGQLIEDVEHTFEEIKPLYEHLHAYVRAKLMNAYPSYISPIGCLPAHLLGDMWGRFWTNLYSLTVPFGQKPNIDVTDAMVDQAWDAQRIFKEAEKFFVSVGLPNMTQGFWENSMLTDPGNVQKAVCHPTAWDLGKGDFRILMCTKVTMDDFLTAHHEMGHIQYDMAYAAQPFLLRNGANEGFHEAVGEIMSLSAATPKHLKSIGLLSPDFQEDNETEINFLLKQALTIVGTLPFTYMLEKWRWMVFKGEIPKDQWMKKWWEMKREIVGVVEPVPHDETYCDPASLFHVSNDYSFIRYYTRTLYQFQFQEALCQAAKHEGPLHKCDISNSTEAGQKLFNMLRLGKSEPWTLALENVVGAKNMNVRPLLNYFEPLFTWLKDQNKNSFVGWSTDWSPYAD

Signal Peptide

Hexa-His tag

TEV Site

ACE2

COV2-2449-LC-ACE2 Fusion

MGWSCIILFLVATATGVHSDIVMTQSPDSLAVSLGERATINCKSSQSLLYTSNNKNYLAWYQQKPGQPPKLLIYWASTRESGVPDRFSGSGSGTDFTLTISSLQAEDVAVYYCQQYYSPPWTFGQGTKVEIKRTVAAPSVFIFPPSDEQLKSGTASVVCLLNNFYPREAKVQWKVDNALQSGNSQESVTEQDSKDSTYSLSSTLTLSKADYEKHKVYACEVTHQGLSSPVTKSFNRGECGGSGS*HHHHHH*ASTGGGSGGPSGQAGAAASE**ENLYFQG**SLFVSNHAYGGSGGEARVSTIEEQAKTFLDKFNHEAEDLFYQSSLASWNYNTNITEENVQNMNNAGDKWSAFLKEQSTLAQMYPLQEIQNLTVKLQLQALQQNGSSVLSEDKSKRLNTILNTMSTIYSTGKVCNPDNPQECLLLEPGLNEIMANSLDYNERLWAWESWRSEVGKQLRPLYEEYVVLKNEMARANHYEDYGDYWRGDYEVNGVDGYDYSRGQLIEDVEHTFEEIKPLYEHLHAYVRAKLMNAYPSYISPIGCLPAHLLGDMWGRFWTNLYSLTVPFGQKPNIDVTDAMVDQAWDAQRIFKEAEKFFVSVGLPNMTQGFWENSMLTDPGNVQKAVCHPTAWDLGKGDFRILMCTKVTMDDFLTAHHEMGHIQYDMAYAAQPFLLRNGANEGFHEAVGEIMSLSAATPKHLKSIGLLSPDFQEDNETEINFLLKQALTIVGTLPFTYMLEKWRWMVFKGEIPKDQWMKKWWEMKREIVGVVEPVPHDETYCDPASLFHVSNDYSFIRYYTRTLYQFQFQEALCQAAKHE

GPLHKCDISNSTEAGQKLFNMLRLGKSEPWTLALENVVGAKNMNVRPLLNYFEPLFTWLKDQNKNSFVGWSTDWSPYAD

Signal Peptide

Knob/Hole Mutations

CrossMAb Swaps

COV2-2449-HC-Knob

MGWSCIILFLVATATGVHSQVQLVESGGGVVQPGRSLRLSCATSGFTFSSFALHWVRQAPGKGLEWVTVISDDGNNKYYVDSVKGRFTISRDNSKNTLFLQMNSLRVEDTAIYYCARASYNSNWSIGEYFRDWGQGTLVTVSSASTKGPSVFPLAPSSKSTSGGTAALGCLVKDYFPEPVTVSWNSGALTSGVHTFPAVLQSSGLYSLSSVVTVPSSSLGTQTYICNVNHKPSNTKVDKKVEPKSCDKTHTCPPCPAPELLGGPSVFLFPPKPKDTLMISRTPEVTCVVVDVSHEDPEVKFNWYVDGVEVHNAKTKPREEQYNSTYRVVSVLTVLHQDWLNGKEYKCKVSNKALPAPIEKTISKAKGQPREPQVYTLPP***C***RDELTKNQVSL***W***CLVKGFYPSDIAVEWESNGQPENNYKTTPPVLDSDGSFFLYSKLTVDKSRWQQGNVFSCSVMHEALHNHYTQKSLSLSPGK

CV10-LC-CrossMAb

MGWSCIILFLVATATGVHSEIVLTQSPGTLSLSPGERATLSCRASQSVSSIYLAWYQQKPGQAPRLLIYGASSRATGIPDRFSGSGSGTDFTLTISRLEPEDFAVYYCQQYAGSPWTFGQ

GTKVEIKSSASTKGPSVFPLAPSSKSTSGGTAALGCLVKDYFPEPVTVSWNSGALTSGVHTFPAVLQSSGLYSLSSVVTVPSSSLGTQTYICNVNHKPSNTKVDKKVEPKSC

CV10-HC-CrossMAb-Hole

MGWSCIILFLVATATGVHSQVQLQESGPGLVKPSETLSLTCNVSGGSISSYYWSWIRQPPGKGLEWIGYIYYSGSTNYNPSLKSRVTISVDTSKNQFSLKLSSVTAADTAVYYCARGFDYWGQGTLVTVSSASVAAPSVFIFPPSDEQLKSGTASVVCLLNNFYPREAKVQWKVDNALQSGNSQESVTEQDSKDSTYSLSSTLTLSKADYEKHKVYACEVTHQGLSSPVTKSFNRGECDKTHTCPPCPAPELLGGPSVFLFPPKPKDTLMISRTPEVTCVVVDVSHEDPEVKFNWYVDGVEVHNAKTKPREEQYNSTYRVVSVLTVLHQDWLNGKEYKCKVSNKALPAPIEKTISKAKGQPREPQV***C***TLPPSRDELTKNQVSL***S***C***A***VKGFYPSDIAVEWESNGQPENNYKTTPPVLDSDGSFFL***V***SKLTVDKSRWQQGNVFSCSVMHEALHNHYTQKSLSLSPGK

Tables:

Supplementary Table 1 – live virus neutralization

Inhibitor	Wuhan-1 50% CPE nM	BA.1 50% CPE nM	BA.2 50% CPE nM
scFv-based ReconnAbs			
CV10	8.1	29	56
CV27	3.3	306	211
2449	10	101	57
IgG-based ReconnAb			
CrossMab	21	171	106
CrossMAb + TEV	375	346	>500.00

Scale	
<200nM	>200nM

Supplementary Table 1 – IC_{50} values from a limiting dilution live SARS-CoV-2 neutralization assay shows ReconnAbs neutralize live viruses as expected. Cytopathic effect (CPE) was scored as binary, either infection or no infection and IC_{50} was calculated using the Spearman-Karber method. Data presented is the average of 3 assays, if one assay was above the limit of detection, the average was computed assuming the limit of detection was the value.
