# Supplementary material for: Converting non-neutralizing SARS-CoV-2 antibodies into broad-spectrum inhibitors
Source: Nat Chem Biol. 2022 Sep 8;18(11):1270–6. doi: 10.1038/s41589-022-01140-1 (PMC9596371; doi:10.1038/s41589-022-01140-1)
Supplement: Supplementary file 2 — Reporting Summary [file 41589_2022_1140_MOESM2_ESM.pdf]

nature research

Corresponding author(s): Peter Kim

Last updated by author(s): Apr 14, 2022

Reporting Summary

Nature Research wishes to improve the reproducibility of the work that we publish. This form provides structure for consistency and transparency in reporting. For further information on Nature Research policies, see our Editorial Policies and the Editorial Policy Checklist.

Statistics

For all statistical analyses, confirm that the following items are present in the figure legend, table legend, main text, or Methods section.

n/a	Confirmed	
☐	☒	The exact sample size (*n*) for each experimental group/condition, given as a discrete number and unit of measurement
☐	☒	A statement on whether measurements were taken from distinct samples or whether the same sample was measured repeatedly
☒	☐	The statistical test(s) used AND whether they are one- or two-sided<br>*Only common tests should be described solely by name; describe more complex techniques in the Methods section.*
☒	☐	A description of all covariates tested
☒	☐	A description of any assumptions or corrections, such as tests of normality and adjustment for multiple comparisons
☐	☒	A full description of the statistical parameters including central tendency (e.g. means) or other basic estimates (e.g. regression coefficient) AND variation (e.g. standard deviation) or associated estimates of uncertainty (e.g. confidence intervals)
☒	☐	For null hypothesis testing, the test statistic (e.g. *F*, *t*, *r*) with confidence intervals, effect sizes, degrees of freedom and *P* value noted<br>*Give P values as exact values whenever suitable.*
☒	☐	For Bayesian analysis, information on the choice of priors and Markov chain Monte Carlo settings
☒	☐	For hierarchical and complex designs, identification of the appropriate level for tests and full reporting of outcomes
☒	☐	Estimates of effect sizes (e.g. Cohen's *d*, Pearson's *r*), indicating how they were calculated

Our web collection on statistics for biologists contains articles on many of the points above.

Software and code

Policy information about availability of computer code

Data collection	For data collection, we used the following commercial and public software applications: Octet System Data Analysis Software version 9.0.0.15 for biolayer interferometry data acquisition, Gen5 2.09 for neutralization luminescence data acquisition on ELx 405 BioTek reader,
Data analysis	Lineage data was analyzed using Geneious Prime 2022.0.1 and all other data was analyzed in Prism 9 (Version 9.3.1), SnapGene software (version 6.0.2), Fiji (version 1) using Image J (version 1.51a) were used to analyze dot blots, linages were made using Geneious Prime 2022.0.1, Conservation analysis was done using the Consurf Server (version 1, consurf.tau.ac.il/), protein structure visualization was done using Pymol (Version 2.3.4), BD CSampler Plus software – version 1.0.34.1 was used for flow analysis.

For manuscripts utilizing custom algorithms or software that are central to the research but not yet described in published literature, software must be made available to editors and reviewers. We strongly encourage code deposition in a community repository (e.g. GitHub). See the Nature Research guidelines for submitting code & software for further information.

Data

Policy information about availability of data

All manuscripts must include a data availability statement. This statement should provide the following information, where applicable:

- Accession codes, unique identifiers, or web links for publicly available datasets
- A list of figures that have associated raw data
- A description of any restrictions on data availability

Antibody sequences were obtained from the CoV-AbDab and coronavirus spike protein alignment sequences from UniRef90. All antibody sequences examined, alignments and phylogenetic trees used are available on Dryad at link https://doi.org/10.7272/Q68S4N53. Raw data is plotted as shown or included as tables.

Field-specific reporting

Please select the one below that is the best fit for your research. If you are not sure, read the appropriate sections before making your selection.

☒ Life sciences ☐ Behavioural & social sciences ☐ Ecological, evolutionary & environmental sciences

For a reference copy of the document with all sections, see nature.com/documents/nr-reporting-summary-flat.pdf

Life sciences study design

All studies must disclose on these points even when the disclosure is negative.

Sample size	For choosing the number of scFvs to include in the yeast library we selected one from at least each group of non-RBD clusters containing 4 or more sequences. The resulting library is described in detail in the methods. The scFv antibody variants to screen for our initial ReconnAb studies were selected such that one antibody from each distinct epitope was included, as described in the text.
Data exclusions	No data were excluded from the analyses.
Replication	All neutralization experiments were replicated at least 2 times, each in duplicate measurements.
Randomization	Samples were allocated in numerical order.
Blinding	Investigators were not blinded to experimental conditions.

Reporting for specific materials, systems and methods

We require information from authors about some types of materials, experimental systems and methods used in many studies. Here, indicate whether each material, system or method listed is relevant to your study. If you are not sure if a list item applies to your research, read the appropriate section before selecting a response.

Materials & experimental systems

n/a	Involved in the study
☐	☒ Antibodies
☐	☒ Eukaryotic cell lines
☒	☐ Palaeontology and archaeology
☒	☐ Animals and other organisms
☒	☐ Human research participants
☒	☐ Clinical data
☒	☐ Dual use research of concern

Methods

n/a	Involved in the study
☒	☐ ChIP-seq
☐	☒ Flow cytometry
☒	☐ MRI-based neuroimaging

Antibodies

Antibodies used	Antibody wildtype sequences for the variable regions were identified from the CoV-AbDab and cloned accordingly, the sequence of the relevant antibodies are provided – other sequences are available already in the CoV-AbDab. Commerical antibodies included anti-mouse IgG1 (Abcam), anti-hexa His antibody (mouse IgG1, BioLegend)
Validation	Anti-mouse IgG1 antibody was isolated from antiserum which was solid phase adsorbed to ensure subclass specificity. The sequence of the protein flanking the poly His tag does not influence antibody binding of the anti-hexa His antibody from BioLegend. g-blocks encoding the scFv plasmids were ordered from Twist biosciences where they are sequence confirmed. A subset were additionally confirmed in our experiments. All ReconnAb proteins described were separately sequence confirmed across the full variable region using sequetech.

Eukaryotic cell lines

Policy information about cell lines

Cell line source(s)	Expi293F cells were obtained from ThermoFisher (catalog number A14527). HeLa-ACE2-TMPRSS2 cells were obtained from the Jesse Bloom Laboratory (Fred Hutch). The Vero E6-TMPRSS2-T2A-ACE2 are from BEI obtained from Raul Andino's lab, they are regularly test (quarterly) for mycoplasma.
Authentication	None of the cell lines were authenticated.
Mycoplasma contamination	All cell lines tested negative for mycoplasma contamination.

Commonly misidentified lines (See ICLAC register) | None

Flow Cytometry

Plots

Confirm that:

☒ The axis labels state the marker and fluorochrome used (e.g. CD4-FITC).

☒ The axis scales are clearly visible. Include numbers along axes only for bottom left plot of group (a 'group' is an analysis of identical markers).

☒ All plots are contour plots with outliers or pseudocolor plots.

☒ A numerical value for number of cells or percentage (with statistics) is provided.

Methodology

Sample preparation: Described in detail in the methods, yeast were stained with biotinylated baits conjugated to streptavidin 647 and anti-c-myc tag FITC. For FACS the yeast library was incubated with 125 nM of tetrameric SARS-CoV-1 and 1 µl of anti-c-myc FITC (Miltenyi) for 1 hour. Samples were then washed 2x with PBSM and then resuspended in 50 µl PBSM. These libraries were then sorted on an FACSAria IIu using the Stanford FACS Facility (Stanford CA).

Instrument: Accuri C6 flow cytometer, FACSAria IIu

Software: BD CSampler Plus software – version 1.0.34.1

Cell population abundance: Abundance is shown in the manuscript. ~21% of all yeast were positive.

Gating strategy: Gates were set such that ~.5% of yeast were antigen positive in the streptavidin alone control.

☒ Tick this box to confirm that a figure exemplifying the gating strategy is provided in the Supplementary Information.
